# Degradation of p0071 and p120-catenin during adherens junction disassembly by *Leptospira interrogans*

**DOI:** 10.3389/fcimb.2023.1228051

**Published:** 2023-09-15

**Authors:** Romina Tokumon, Isabel Sebastián, Bruno M. Humbel, Nobuhiko Okura, Hidenori Yamanaka, Tetsu Yamashiro, Claudia Toma

**Affiliations:** ^1^ Department of Bacteriology, Graduate School of Medicine, University of the Ryukyus, Okinawa, Japan; ^2^ Provost Office, Okinawa Institute of Science and Technology Graduate University, Okinawa, Japan; ^3^ Microscopy Center, Universidade Federal de Minas Gerais, Belo Horizonte, MG, Brazil; ^4^ Department of Cell Biology and Neuroscience, Juntendo University Graduate School of Medicine, Tokyo, Japan; ^5^ Department of Molecular Anatomy, Graduate School of Medicine, University of the Ryukyus, Okinawa, Japan; ^6^ Environmental Technology Department, Chemicals Evaluation and Research Institute, Saitama, Japan

**Keywords:** *Leptospira interrogans*, adherens junction, epithelial cell, p0071, p120-catenin, E-cadherin

## Abstract

*Leptospira interrogans* disseminates hematogenously to reach the target organs by disrupting epithelial adherens junctions (AJs), thus causing leptospirosis, which is a globally neglected zoonotic disease. *L. interrogans* induces E-cadherin (E-cad) endocytosis and cytoskeletal rearrangement during AJ disassembly, but the detailed mechanism remains unknown. Elucidation of AJ disassembly mechanisms will guide new approaches to developing vaccines and diagnostic methods. In this study, we combine proteomic and imaging analysis with chemical inhibition studies to demonstrate that disrupting the AJs of renal proximal tubule epithelial cells involves the degradation of two armadillo repeat-containing proteins, p0071 and p120-catenin, that stabilize E-cad at the plasma membrane. Combining proteasomal and lysosomal inhibitors substantially prevented p120-catenin degradation, and monolayer integrity destruction without preventing p0071 proteolysis. In contrast, the pan-caspase inhibitor Z-VAD-FMK inhibited p0071 proteolysis and displacement of both armadillo repeat-containing proteins from the cell-cell junctions. Our results show that *L. interrogans* induces p120-catenin and p0071 degradation, which mutually regulates E-cad stability by co-opting multiple cellular degradation pathways. This strategy may allow *L. interrogans* to disassemble AJs and disseminate through the body efficiently.

## 1 Introduction

During acute infections, bacterial pathogens can destroy the body’s physical barriers to facilitate adherence and toxin uptake or to take advantage of an opened cell-cell junction and disseminate throughout the body. Opening these junctions requires the disassembly of the apical junctional complexes (AJCs) (Huber, 2020). The AJCs comprise the junctions at the lateral sides of the cell membrane from the apical to the basal side: tight junctions, adherens junctions (AJ), gap junctions, and desmosomes. AJ stability with E-cadherin (E-cad) is critical for maintaining AJC organization and epithelial tissue barrier function (Díaz-Díaz et al., 2020). E-cad is a transmembrane protein with extra- and intracellular domains organized into nanoscale clusters distributed throughout cell-cell junctions. E-cad clustering can be achieved through adhesive (*trans*) interactions between ectodomains presented on neighboring cell surfaces and lateral (*cis*) interactions between E-cads presented on the same cell surface (Yap et al., 2015). The classical catenins (ctns), α- and β-ctn, bridge the cadherin cytoplasmic domain to the underlying actin cytoskeleton. Thus, E-cad is a master organizer of the epithelial phenotype (Takeichi, 2018). Cadherin stabilization (E-, vascular endothelial-, and neural-cad) at the membrane is a common function of a subfamily of armadillo proteins that comprises p120-catenin (p120-ctn), p0071, armadillo repeat gene deleted in velo-cardio-facial syndrome, and δ-ctn/neural plakophilin-related armadillo repeat protein (Keil et al., 2013). Armadillo proteins are characterized by a central domain that comprises a series of approximately 40–45 amino acid-long repeated sequence motifs, providing a versatile scaffold for protein-protein interactions. Besides the association with the cytoplasmic domain of cadherin superfamily members, some of the properties that are shared amongst ctn-proteins are their cytoplasmic and nuclear localization, and their interaction with small GTPases to regulate the cytoskeletal organization and actin dynamics (Davis et al., 2003).

Pathogenic *Leptospira* spp. colonize the renal proximal tubules of reservoir hosts and are shed in the urine, contaminating the environment (Yamaguchi et al., 2018; Sato et al., 2022). Their ability to survive in water and soil, and infect a wide range of host species, including humans and wild and domestic animals, has made leptospirosis a zoonotic disease of increasing importance worldwide (Picardeau, 2017; Bierque et al., 2020). Upon infection, leptospires can traverse tissue barriers to rapidly disseminate to all organs, including the eyes and brain (Coburn et al., 2021). *In vitro* infection of cell monolayers showed that translocation of leptospires can occur via trans- and paracellular pathways (Barocchi et al., 2002; Sebastián et al., 2021), whereas animal infection models have shown leptospires primarily in the intercellular spaces of the liver, kidneys, intestines, and lungs (Marshall, 1976; Miyahara et al., 2014; Nikaido et al., 2019; Inamasu et al., 2022). AJs are the primary target during AJC disassembly induced by *L. interrogans* in epithelial and endothelial cells (Sato and Coburn, 2017; Sebastián et al., 2021). The extracellular domain of E-cad is essential for the maintenance of tissues integrity and prevention of cell migration during pathogenic bacterial infections. Thus, E-cad extracellular domain shedding is a bacterial strategy to disrupt intercellular adhesion (Hoy et al., 2010; Hsu et al., 2021). During leptospiral renal proximal tubule epithelial cell (RPTEC) infection, non-pathogenic *L. biflexa* and pathogenic *L. interrogans* induce E-cad extracellular domain shedding; however, only pathogenic leptospires can induce E-cad endocytosis and AJ disassembly (Sebastián et al., 2021). The E-cad-juxtamembrane domain (JMD) of the intracellular region binding to p120-ctn is also required to maintain the mechanical integrity of tissues (Vu et al., 2021). Thus, the leptospiral AJ disassembly may need additional mechanisms to E-cad ectodomain shedding in order to efficiently disrupt lateral E-cad clusters and reduce cell-cell adhesion during paracellular transmigration. Elucidation of leptospiral AJ disassembly mechanisms will guide new approaches to the development of vaccines and diagnostic methods.

In eukaryotic cells, proteases can cleave proteins at specific site(s) to generate peptides in the so-called “limited proteolysis” (Sriram et al., 2011). Two major intracellular protein degradation pathways are involved in digestive proteolysis: autophagy and the ubiquitin-proteasome system (UPS) (Minina et al., 2017). Autophagy is the formation of double-membrane structures termed as autophagosomes, which fuse with lysosomes to degrade their contents (Kocaturk and Gozuacik, 2018). Proteasomes are organelles that degrade proteins tagged with ubiquitin, a polypeptide of 76 amino acids that can be covalently attached to substrate proteins by a ubiquitin ligase protein (E3). Ubiquitination is an essential post-translational modification that regulates protein stability, receptor internalization, and protein-protein interactions, and can be involved in the degradation of cadherins and their interacting proteins to disrupt the epithelial and endothelial barrier (Cai et al., 2018). Ubiquitination can also mark invading bacteria, promoting their killing via UPS or autophagolysosome-mediated degradation pathways (Fiskin et al., 2016). Thus, some bacterial pathogens have evolved survival strategies for hijacking this system to achieve successful infection (Ashida and Sasakawa, 2017). Other pathogens can activate intracellular host proteases or promote their expression and secretion to benefit bacteria in a tightly regulated spatiotemporal manner to ensure a safely replication niche and efficient dissemination (Bergounioux et al., 2012). Bacterial proteases can also directly target AJC proteins for degradation, such as tight junction-membrane protein claudins in *Aeromonas* and *Campylobacter* infections (Sharafutdinov et al., 2020; Ueda et al., 2022). Several leptospiral proteases capable of degrading extracellular matrix or complement factors were identified by experimental and *in silico* studies (Daroz et al., 2020; Daroz et al., 2021; Courrol et al., 2022). However, a specific protease for degrading AJC proteins has not been identified yet.

In a prior study, we developed a leptospiral-cell infection model using RPTECs immortalized by ectopic expression of the telomerase reverse transcriptase catalytic subunit, which shows the morphological and functional properties of primary renal epithelial cells (Wieser et al., 2008; Sebastián et al., 2021). In this study, we used this model to clarify the strategies used by *L. interrogans* to disassemble the AJs and destroy the monolayer integrity. This is the first study to provide evidence that *L. interrogans* hijacks eukaryotic proteolytic systems to achieve successful infection.

## 2 Materials and methods

### 2.1 Bacterial cultures and strains

*L. interrogans* serovar Manilae strain UP-MMC-NIID (Koizumi and Watanabe, 2004) that was passaged no more than three times to maintain reproducible virulence (Toma et al., 2014), and *L. biflexa* serovar Patoc strain Patoc 1 were routinely stationary cultured in Ellinghausen-McCullough-Johnson-Harris (EMJH) broth at 30°C. *L. interrogans* was diluted 20-fold, and *L. biflexa* was diluted 40-fold in fresh EMJH and cultured for 3 days with shaking at 30°C for cell infection experiments.

### 2.2 Cell culture

RPTEC/TERT1 (American Type Culture Collection, ATCC^®^ CRL-4031™) cells were grown in Dulbecco’s Modified Eagle Medium/Nutrient Mixture F-12 Ham (DMEM/F-12 Ham; Sigma-Aldrich, St. Louis, MI, USA) supplemented with 5 pM triiodothyronine, 10 ng/mL recombinant human epidermal growth factor, 3.5 μg/mL ascorbic acid, 5 μg/mL transferrin, 5 μg/mL insulin, 8.65 ng/mL sodium selenite and 100 μg/mL G418. The cells were seeded at a density of 1 × 10^6^ cells/well in polyethylene terephthalate hanging cell culture inserts with a pore size of 3 μm (Falcon^®^; Corning, New York, NY, USA) in the upper chamber of a Falcon^®^ Companion six-well tissue culture plate (Corning). Cells were maintained in a humidified incubator at 37°C and 5% CO_2_ for 7 to 10 days after reaching confluence to allow monolayer maturation, and the medium was exchanged every 2 days. The transepithelial electrical resistance (TEER) of the monolayers was measured using a Millicell-ERS cell resistance indicator (MilliporeSigma, Burlington, MA, USA). After subtraction of the value of a cell-free filter (blank), the mean TEER value was expressed as Ωcm^2^. The TEERs of cells before infection were designated as the baseline values. The percentage TEER, relative to the baseline value, was calculated using the following formula: (TEER of experimental wells/baseline TEER of experimental wells) ×100%.

### 2.3 Cell infection

The cells were infected with leptospires at a multiplicity of infection (MOI) of 100 from the basolateral side containing supplement-free DMEM/F-12 Ham medium. In the experiments involving the use of inhibitors, the inhibitors were added 30 min before infection. The inhibitors used were 30 μM dynasore (Sigma-Aldrich), 100 nM bafilomycin A1 (Bioviotica, Liestal, Switzerland), 50 μM chloroquine (Sigma-Aldrich), 10 μM MG-132 (Sigma-Aldrich), 200 nM bortezomib (FUJIFILM Wako, Osaka, Japan), 20 μM benzyloxycarbonyl-Val-Ala-Asp(OMe)-fluoromethylketone (Z-VAD-FMK, a pan-caspase inhibitor) (Tocris Bioscience, Bristol, UK), 20 μM benzyloxycarbonyl-Asp(OMe)-Glu(OMe)-Val-Asp(OMe)-fluoromethylketone (Z-DEVD-FMK, a caspase-3-specific inhibitor) (Tocris Bioscience), or 50 μM MDL-28170 (calpain inhibitor III) (Tokyo Chemical Industry, Tokyo, Japan). Cells were then incubated at 37°C and 5% CO_2_ during infection and lysed for proteome analysis and immunoblotting, or fixed for proximity ligation assay (PLA), immunostaining, and electron microscopy analysis.

### 2.4 Proteome analysis

Liquid chromatography with tandem mass spectrometry (LC-MS/MS) analyses were performed using Nano LC (UltiMate^®^ 3000; Dionex, Sunnyvale, CA, USA) coupled with Q Exactive plus (Thermo Scientific, Waltham, MA, USA) (Yates et al., 1995). Instrument operation and data acquisition were performed using Xcalibur Software (version 3.1.66.1; Thermo Fisher Scientific, Waltham, MA, USA). Samples were reduced and alkylated as previously described (Peng et al., 2011). Proteins were diluted 3-fold with 50 mM ammonium bicarbonate (pH 8.5) and digested with trypsin at an enzyme-to-substrate ratio of 1:50 (wt/wt, modified sequencing grade, Promega, Madison, WI, USA) for 16 h at 30°C. Digested peptides were directly injected. The peptides were separated on a 500 × 0.075 mm capillary reversed-phase column (CERI, Tokyo, Japan) at a flow rate of 500 nL/min and a column temperature of 60°C. The mobile phase comprised water with 0.1% (v/v) formic acid (eluent A) and 80% acetonitrile with 0.1% (v/v) formic acid (eluent B). The peptides were separated by a linear gradient of up to 35% eluent B in 340 min for a 6 h gradient run.

The Q Exactive plus (Thermo Fisher Scientific) was operated in data-dependent (dd) mode with full scans acquired at a resolution of 35,000 at 350 m/z and with dd-MS/MS scans acquired at a resolution of 17,500. The mass spectrometer was operated in positive mode in the scan range of 350 –1,800 m/z. Fixed first m/z is 150 in dd-MS/MS scans. The 10 most abundant isotope patterns with a charge ≥2 from the survey scan were selected using an isolation window of 1.6 m/z. The maximum ion injection times for the full scan and the dd-MS/MS scans were 20 ms and 100 ms, respectively, and the automatic gain control for the full and the dd-MS/MS scans were 3E6 and 1E5, respectively. Repeat sequencing of peptides was kept to a minimum by the dynamic exclusion of the sequenced peptides for 20 s.

The database search was performed against the UniProt database of humans (Taxonomy ID:9606, released in June 2021) and NCBInr database of *L. interrogans* serovar Manilae (Taxonomy ID: 214675, released on 06/29/2021) using Proteome Discoverer version 2.5 (PD2.5, Thermo Fisher Scientific) with the MASCOT (Matrix Science, Boston, MA, USA) and Sequest (Thermo Fisher Scientific) search engine software (Mann and Wilm, 1994; Perkins et al., 1999; Shalit et al., 2015). Search parameters were as follows: fixed modifications, carbamidomethyl; variable modifications, oxidation (M); missed cleavages, up to 1; monoisotopic peptide tolerance, 10 ppm; and MS/MS tolerance, 0.6 Da. The PD2.5 search result files were uploaded into Scaffold (version 5.0.1, Proteome Software Inc., Portland, OR, USA) for protein identification and label-free quantitation based on an intensity-based absolute quantification value (Spinelli et al., 2012). The two LC-MS/MS technical replicates (n = 2) of each group were combined in Scaffold. The in-built normalization function and Student’s t-test was used to calculate the fold changes and *p-*values of protein abundances between two groups (i.e., non-infected group and infected group at 4 h or 24 h post-infection (p.i.)). Proteins that were detected with 99.0% probability (protein FDR = 0.2%) assigned by ProteinProphet (Keller et al., 2002) and containing at least two peptides that were detected with Mascot Threshold were considered positive identifications and quantified. The proteomics dataset has been deposited to the ProteomeXchange and jPOST (Okuda et al., 2017) with the dataset identifiers PXD040838 and JPST002081, respectively.

### 2.5 PLA

**Figure 1:**
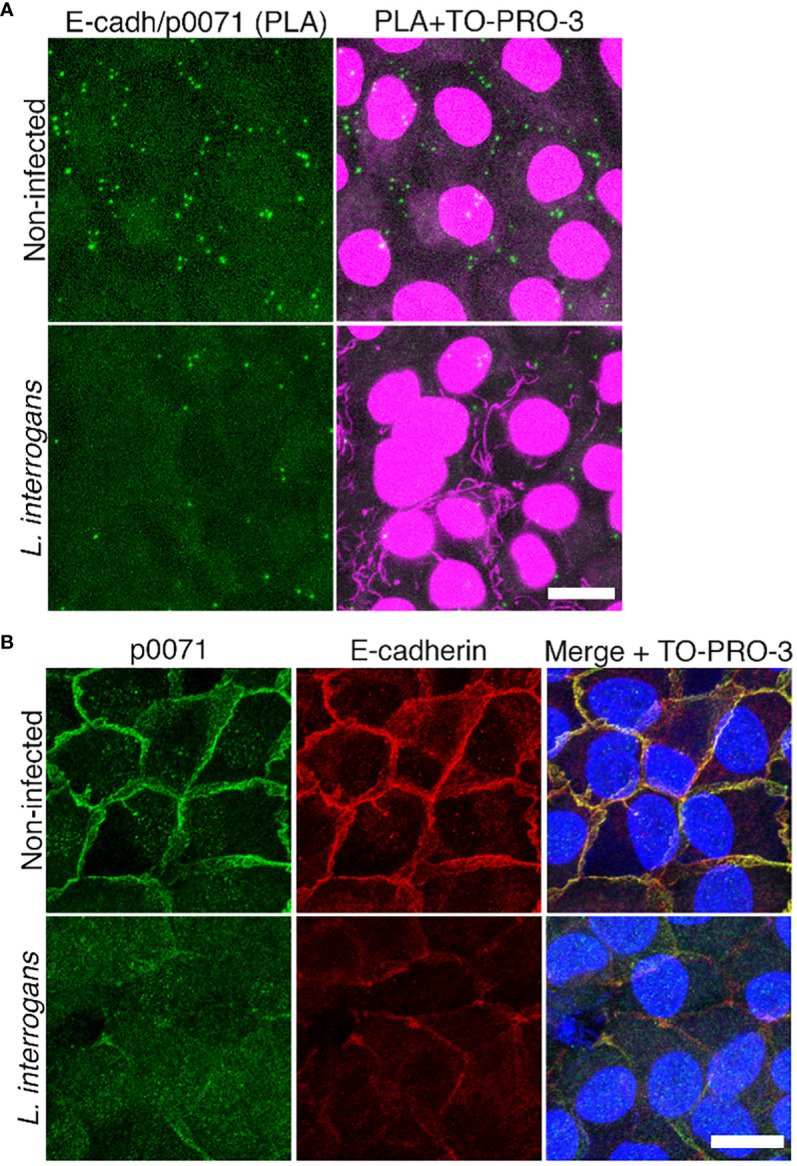
Analysis of E-cadherin and p0071 interaction and localization. **(A)** *In situ* proximity ligation assay (PLA) in non-infected and *L. interrogans*-infected RPTECs. Protein interaction was visualized as green “spots.” Cell nuclei and leptospiral DNA were counterstained with TO-PRO-3 (magenta). Scale bar: 10 μm. **(B)** Representative confocal images of non-infected and *L. interrogans*-infected RPTECs. Cells were fixed with methanol and processed for immunostaining at 24 h p.i. p0071 was stained with an Alexa Fluor 488-labeled antibody (green), and E-cadherin with a Cy3-labeled antibody (red). The cell nuclei were counterstained with TO-PRO-3 (blue). Scale bar: 10 μm.

For the PLA (**Figure 1**), non-infected or infected cells were methanol-fixed, permeabilized, and blocked with blocking buffer (5% bovine serum albumin (BSA), 1% Triton X-100 in Tris-buffered saline (TBS; 50 mM Tris and 150 mM NaCl (pH 7.4)) for 15 min. The antibodies used for analyses were rabbit monoclonal anti-Ecad (1:50; #3195, Cell Signaling Technology (CST) Danvers, MA, USA) and multi-epitope cocktail mouse monoclonal anti-p0071 (ready-to-use, #651166, ProGen, Baden-Wurttemberg, Germany). PLA was conducted using the DuoLink PLA kit (Sigma-Aldrich) with Detection Reagents Green by following the manufacturer’s protocol.

### 2.6 Immunoblotting

**Figure 2:**
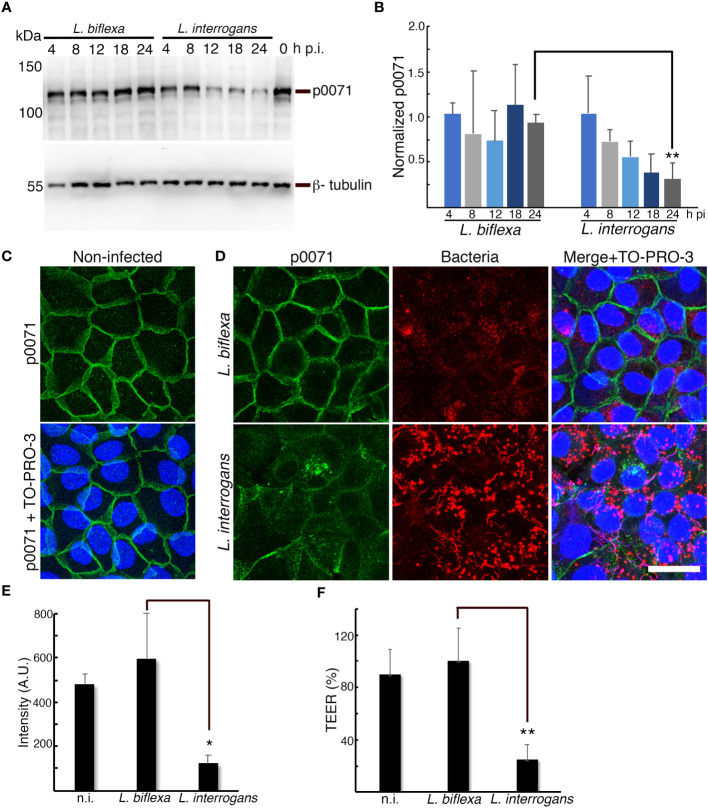
p0071 is decreased and displaced from the AJs in *L. interrogans-*infected RPTECs. ***(*A*)*** RPTECs were infected either with *L. biflexa* or *L. interrogans* and whole cell lysates were subjected to western blotting with anti-p0071 or β-tubulin at the indicated time post- infection (p.i.). **(B)** Normalized levels of p0071. **(C, D)** Representative confocal images of non-infected **(C)**, or *L. biflexa*- or *L. interrogans*-infected RPTECs at 24 h p.i. **(D)**. Cells were fixed with methanol and processed for immunostaining: p0071 was stained with an Alexa Fluor 488-labeled antibody (green), and bacteria were stained with a Cy3-labeled antibody (red). The cell nuclei were counterstained with TO-PRO-3 (blue). Scale bar: 25 μm. **(E)** Quantification of fluorescence intensity at cell-cell junctions. A.U., arbitrary units. **(F)** Transepithelial electrical resistance (TEER) measurements to evaluate monolayer integrity at 24 h p.i. Each bar represents the mean ± standard deviation of three independent experiments. *p <0.05 and **p <0.01.

For initial analysis of infected cells, RPTECs were lysed with a mild lysis buffer: 20 mM Tris-HCl (pH 7.4), 150 mM NaCl, and 0.1% Nonident P-40, supplemented with a protease inhibitor cocktail (Roche, Basel, Switzerland). The cell lysates were collected with a cell scrapper for centrifugation, and supernatants were processed for immunoblotting (**Figure 2**). For further analysis (all other immunoblots), cell lysates were obtained with RIPA buffer (Nacalai, Kyoto, Japan) [50 mM Tris-HCl buffer (pH7.6), 150 mM NaCl, 1% Nonidet P40, 0.5% sodium deoxycholate, and 0.1% sodium dodecyl sulphate (SDS)], supplemented with a protease inhibitor cocktail (Roche). Cell lysates obtained with RIPA buffer were analyzed without centrifugation to consider those proteins that are in the insoluble fraction owing to their association with the cytoskeletal proteins network (Sebastián et al., 2021). Cell lysates were mixed with Laemmli sample buffer (Laemmli, 1970) and heated for 10 min at 100°C. The RIPA cell lysates were sonicated for a total of 3 min (5s sonication with 5s intervals), and the samples were separated using 7.5% SDS-gel electrophoresis and further processed for immunoblots. Amersham ImageQuant 800 Imaging System (GE Healthcare, Chicago, IL, USA) was used to visualize the protein bands.

Primary antibodies used for immunoblotting included rabbit polyclonal anti-p0071 (1:1000, #A304-649AA, Bethyl Laboratories, Montgomery, TX, USA); mouse monoclonal antibodies: anti-p120-ctn (1:2500; sc-23872, Santa Cruz Biotechnology, Inc., Dallas, TX, USA), anti-α-catenin (1:1000; sc-9988) and anti-ubiquitin (1:5000, sc-8017); and rabbit monoclonal antibodies: anti-β-tubulin (1:1000; CST#2128), anti-E-cad (1:1000; CST#3195), anti-plakoglobin (1:1000; CST#7550), and anti-β-catenin (1:1000; CST#8480). The secondary antibodies were a horseradish peroxidase (HRP)-anti-rabbit antibody (1:10,000; #111-035-144, Jackson ImmunoResearch (JIR), West Grove, PA, USA) and HRP-anti-mouse antibody (1:10,000; JIR#715-005-150).

### 2.7 Immunostainings

RPTECs were fixed in cold methanol for 15 min, permeabilized, and blocked with the same blocking buffer used for PLA for 15 min to analyze the localization of AJC proteins. For the immunostaining of the lysosomal-associated membrane protein-1 (LAMP-1) and for F-actin staining, infected cells were fixed with 2% paraformaldehyde in phosphate-buffered saline for 5 h at 4°C and washed twice with TBS. The filter membranes were detached from the hanging culture inserts before immunostaining.

Antibodies were diluted in TBS containing 1% BSA and 0.1% Triton X-100, except in LAMP-1 immunostainings wherein TBS containing 0.2% saponin and 10% Blocking One (Nacalai) was used as the antibody dilution buffer (Toma and Suzuki, 2020). The primary antibodies for bacteria were diluted rabbit polyclonal anti-*L. interrogans* antibody (1:500; kindly provided by Sharon YAM Villanueva, University of the Philippines) (Villanueva et al., 2014) and anti-*L. biflexa* antibody (1:500; Affinity BioReagents, Golden, CO, USA). RPTEC proteins were immunostained with multi-epitope cocktail mouse monoclonal anti-p0071 (ready-to-use, #651166, ProGen); mouse monoclonal antibodies: anti-p120-ctn (1:50; sc-373751), anti-LAMP1 (1:200; sc-20011), anti-acetylated tubulin (1:100; #T7451, Sigma-Aldrich) and, anti-E-cad (1:50; # 610182, BD Transduction Laboratories, Franklin Lakes, NJ, USA); rabbit monoclonal anti-E-cad (1:50; CST#3195) and anti-p120-ctn (1:300; CST#59854); and rabbit polyclonal anti-p0071 (1:1000, #A304-649AA, Bethyl Laboratories). F-actin was stained with a 1:500 dilution of rhodamine phalloidin (Abcam, Cambridge, UK). Cells were counterstained to label the DNA with a 1:100 dilution of TO-PRO™-3 (Invitrogen, Waltham, MA, USA). Secondary antibodies used for immunofluorescence analysis included anti-rabbit Alexa 488- (1:100; JIR#711-545-152), Cy3- (1:100; JIR#711-165-152), or Alexa 647-conjugated antibody (1:100, JIR#711-605-152); and anti-mouse Alexa 488- (1:100; JIR#715-545-151) or Cy3-conjugated antibody (1:100; JIR#715-165-151). The compiled Z-stack images were acquired using a Leica TCS-SPE confocal laser scanning microscope after mounting with SlowFade™ Diamond Antifade Mountant (Invitrogen) using LEICA LAS AF acquisition software (version 2.6.0.7266, Leica Microsystems CMS GmbH, Germany). Quantification of the fluorescence intensity of immunostained proteins at cell-cell junctions were measured from maximum projections of Z-stacks images using ImageJ software.

### 2.8 Focused ion beam scanning electron microscopy

RPTECs were infected for 16 h and pre-fixed with 2.5% glutaraldehyde in phosphate buffer (0.1 M, pH 7.4), and then post-fixed with 2% osmium tetroxide and 1.5% potassium hexacyanoferrate(II) in water for 1 h. Specimens were stained overnight *en bloc* with a 2% (w/v) uranyl acetate aqueous solution, dehydrated with serially increasing concentrations of ethanol (30–100%), and embedded in epoxy resin according to standard protocol.

Volume microscopy was performed using a focused ion beam scanning electron microscope (Helios 650; FEI Company, Eindhoven, The Netherlands) (Kizilyaprak et al., 2019). Milling was performed with a gallium ion beam at 30 kV and 2.5 nA, and the imaging was done at 1.5 keV, 800 pA, 6144 × 4096-pixel frame size, 6 μs dwell time, with 70 Å pixel size and 200 Å section thickness (Kizilyaprak et al., 2015). After milling at 52°, the fresh surface was tilted to 90° and imaged normally to the electron beam (see accompanying video of Kizilyaprak et al., 2015). The images were aligned with IMOD (Kremer et al., 1996) using the “Align Serial Sections/Blend Montages” function.

### 2.9 Statistical analyses

Band intensities in immunoblots were analyzed using ImageJ software (version 1.53). Reacting protein bands were normalized with β-tubulin as a loading control, and the relative expression level of each protein was calculated by considering the ratio of analyzing protein/β-tubulin in non-infected RPTECs as one. Statistical significance was determined using an unpaired two-tailed Student *t*-test. All the data were presented as the mean of at least three independent determinations per experimental condition. Differences were considered significant at a *p-*value of < 0.05.

## 3 Results

### 3.1 *L. interrogans* infection decreases the multifunctional protein p0071 in RPTECs

*L. interrogans* induces E-cad endocytosis, resulting in AJ disassembly. However, a transcriptomics approach revealed that AJ-associated proteins were not differentially expressed, suggesting that post-transcriptional events are critical during AJ disruption by *L. interrogans* (Sebastián et al., 2021). In this study, to understand the mechanism of AJ disruption, we analyzed the changes in protein profiles in response to *L. interrogans* infection via a proteome analysis of non-infected and infected RPTECs (dataset identifiers in the repositories are described in the Materials and Methods section). Comparing protein profiles at 4 h p.i. and 24 h p.i. indicated that 106 RPTEC proteins were decreased and 47 were increased with a fold change of <0.5 and >2, respectively (**Tables S1**, **S2**). Notably, a multifunctional protein coordinating cell adhesion with cytoskeletal organization, p0071 (also known as plakophilin-4) (Keil and Hatzfeld, 2014), was decreased 50 times at 24 h p.i. (**Table S1**). We first focused on p0071 and analyzed whether it is associated with E-cad in RPTECs as reported in other cell types (Hofmann et al., 2009; Medvetz et al., 2012) using an *in situ* PLA. Non-infected RPTECs showed green spots surrounding the cells, representing p0071/E-cad interactions; these green spots decreased in *L. interrogans*-infected RPTECs (**Figure 1A**). Dual immunofluorescence indicated the co-localization of p0071 and E-cad in non-infected RPTECs which was decreased in *L. interrogans*-infected RPTECs (**Figure 1B**).

In a time course experiment, we quantitatively analyzed p0071 by conducting immunoblotting assays of non-infected and *L. biflexa-* or *L. interrogans*- infected RPTECs. p0071 protein levels gradually decreased in *L. interrogans*- and *L. biflexa*-infected RPTECs until 12 h p.i. However, in *L biflexa*-infected RPTECs, p0071 levels were recovered to basal levels at 24 h p.i. In contrast, in *L. interrogans*-infected RPTECs, p0071 continuously decreased during the course of infection and was significantly decreased at 24 h p.i. (~ 30% of *L. biflexa*-infected RPTECs, *p <*0.01) (**Figures 2A, B**). Immunofluorescence analysis showed that p0071 was localized at AJs in non-infected and *L. biflexa*-infected RPTECs but it was displaced from cell-cell junctions in *L. interrogans*-infected RPTECs at 24 h p.i. (**Figures 2C–E**). Leptospiral immunostaining showed that the bacterial population interacting with epithelial cells was higher in *L. interrogans*-infected RPTECs than in *L. biflexa*-infected RPTECs (**Figure 2D**). The monolayer integrity was disrupted in *L. interrogans*-infected RPTECs as indicated by the evaluation of TEER, which reduced to ~25% of the initial TEER (**Figure 2F**). These results suggest that the p0071 decrease is associated with *L. interrogans* pathogenicity related to AJ disassembly.

### 3.2 *L. interrogans* infection decreases catenin proteins that interact with the E-cad juxtamembrane domain

**Figure 3:**
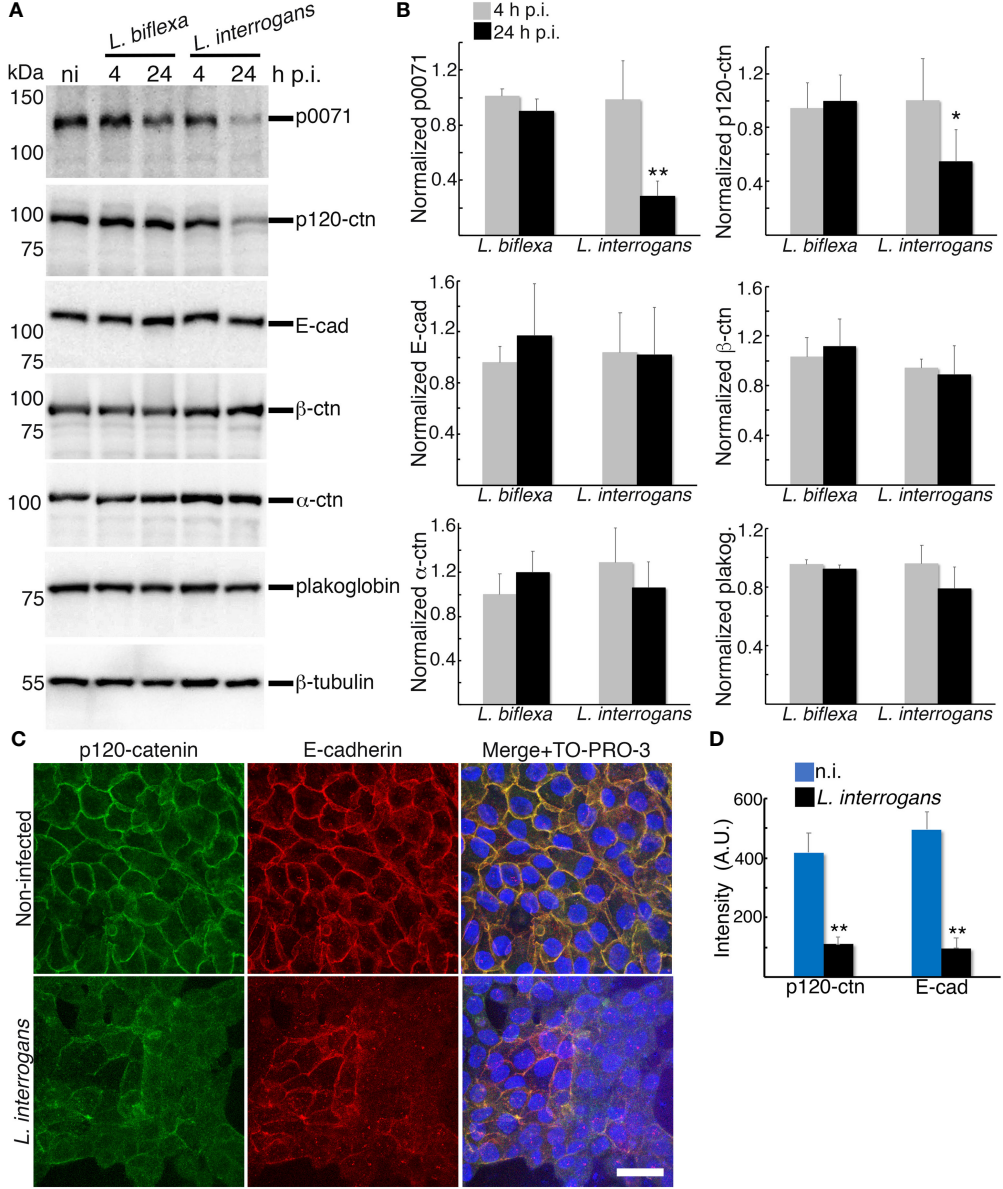
Analysis of proteins that stabilizes the E-cad at the plasma membrane and link E-cad to the cytoskeleton. **(A)** RPTECs were infected either with *L. biflexa* or *L. interrogans* and whole-cell lysates were subjected to western blotting with the indicated antibodies at 4 h and 24 h p.i. **(B)** Normalized levels of AJC-proteins. Significance was tested by comparing the protein levels with that of non-infected RPTECs. **(C)** Representative confocal images of non-infected or *L. interrogans*-infected RPTECs. Cells were fixed with methanol and processed for immunostaining at 24 h p.i. p120-ctn was stained with an Alexa Fluor 488-labeled antibody (green), and E-cad was stained with a Cy3-labeled antibody (red). The cell nuclei were counterstained with TO-PRO-3 (blue). Scale bar: 25 μm. **(D)** Quantification of the fluorescence intensity at cell-cell junctions. A.U., arbitrary units. Each bar represents the mean ± standard deviation of three independent experiments. *p <0.05 and **p <0.01.

Comparison of proteomes at 4 h p.i. and 24 h p.i. showed that, in addition to p0071, three ctns with armadillo domains were decreased at 24 h p.i.: p120-ctn, β-ctn (scaffold proteins of the AJ), and plakoglobin (a desmosomal scaffold protein), with a decrease of 10, 5, and 3 times, respectively (**Table S1**). Among the AJ proteins, E-cad and α-ctn (a protein that links the AJ to the cytoskeleton) also showed a decrease of 10 and 3 times, respectively (**Table S1**). For a more accurate quantitative analysis, we compared these proteins levels in total cellular lysates of *L. biflexa*- and *L. interrogans*-infected RPTECs at 4 h and 24 h p.i. with proteins levels of non-infected RPTECs. The three independent experiments showed that p120-ctn was significantly decreased at 24 h p.i. in *L. interrogans*-infected RPTECs (*p <*0.05), whereas E-cad, β-ctn, α-ctn, and plakoglobin were not decreased (**Figures 3A, B**). Therefore, we ruled out the possibility of E-cad-Hakai-dependent ubiquitination and proteasomal degradation as a trigger event of the *L. interrogans*-induced AJ disassembly (Hartsock and Nelson, 2012). Immunofluorescence analysis showed that *L. interrogans* infection induced significant p120-ctn and E-cad displacement from the AJs (*p <*0.01; **Figures 3C, D**). Dual immunofluorescence analysis of p0071 and plakoblogin at 24 h p.i. showed that, in *L. interrogans*-infected RPTECs, plakoglobin was not significantly displaced from the cell-cell junctions (*p >*0.05; **Figures S1A, B**). These results suggest that *L interrogans* specifically induces mislocalization and decrease in the levels of the p120-ctn sub-family proteins (p0071 and p120-ctn) that interact with the cytoplasmic E-cad JMD to displace the AJ from the plasma membrane.

### 3.3 Inhibition of clathrin-mediated endocytosis prevents p0071 and p120-ctn decrease and their displacement from the AJs

**Figure 4:**
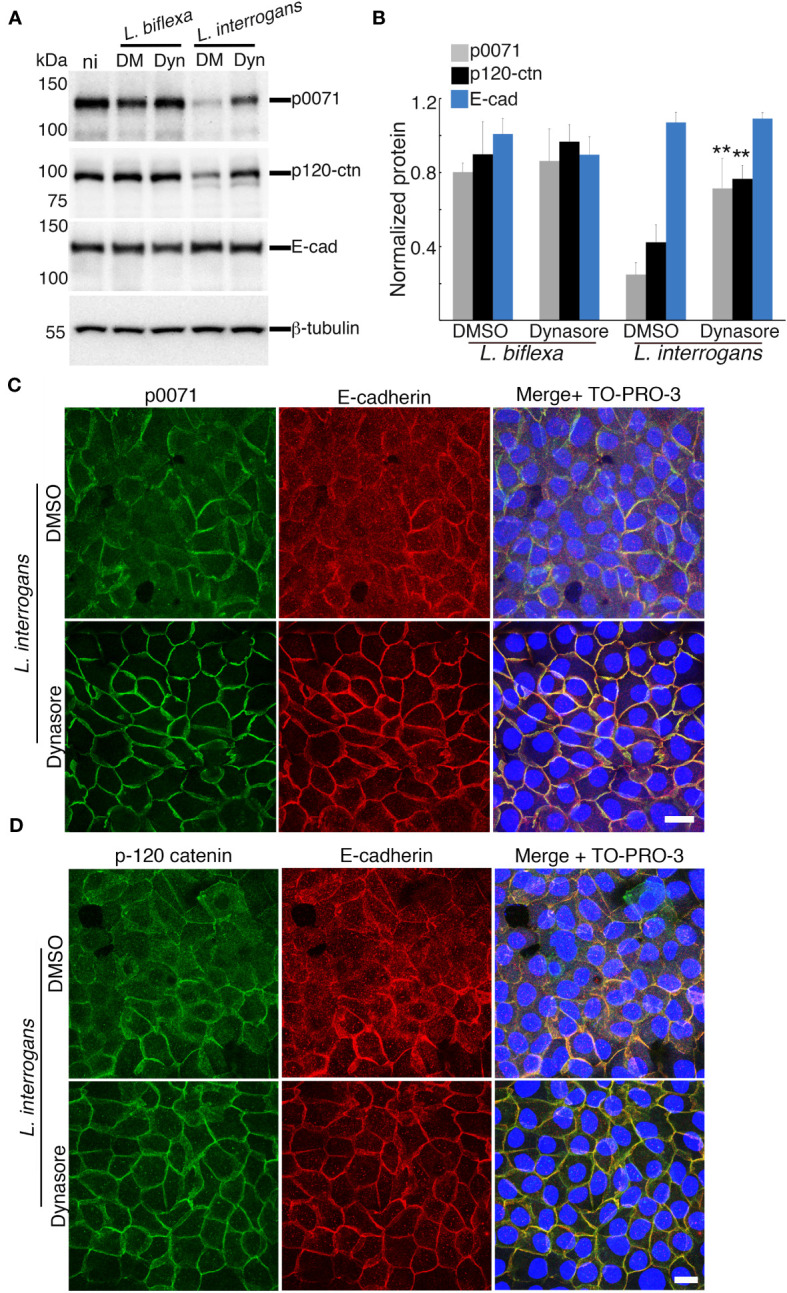
Inhibition of clathrin-mediated endocytosis prevents p0071 and p120-ctn degradation and their displacement from the AJs. Dynasore- or DMSO-pre-treated RPTECs were infected with either *L. biflexa* or *L. interrogans*. **(A)** Whole-cell lysates were subjected to western blotting with the indicated antibodies at 24 h p.i. **(B)** Normalized levels of AJ-proteins. Each bar represents the mean ± standard deviation of three independent experiments. **p <0.01. Significance was tested by comparing the protein levels with that of DMSO-treated infected RPTECs. **(C, D)** *L. interrogans*-infected RPTECs were immunostained with anti-p0071 **(C)** or p120-ctn **(D)** antibodies and then visualized with Alexa Fluor 488-conjugated secondary antibodies (green). E-cad was detected with a Cy3-labeled antibody (red) and the cell nuclei were detected with TO-PRO-3 (blue). Scale bar: 10 μm.

We hypothesized that p0071 and p120-ctn degradation may be the trigger and upstream event of the clathrin-mediated endocytosis of E-cad found in our previous study (Sebastián et al., 2021). Dynasore-pre-treated RPTECs were infected with *L. biflexa* or *L. interrogans* and total cell lysates were analyzed via immunoblotting to evaluate this hypothesis. Inhibition of endocytosis with dynasore significantly prevented p0071 and p120-catenin degradation (*p <*0.01) (**Figures 4A, B**). Dynasore treatment did not affect localization of p0071, p120-ctn, and E-cad in non-infected RPTECs (**Figure S2**). Subsequently, we analyzed the effect of dynasore pre-treatment in infected RPTECs by immunofluorescence and found that dynasore prevented the displacement of p0071 and p120-ctn from the AJs (**Figures 4C, D**). These results suggest that p0071 and p120-ctn degradation does not trigger AJ disassembly and an endocytic event precedes p0071 and p120-ctn degradation.

### 3.4 Lysosomal and proteasomal inhibition prevents p120-ctn degradation but not p0071 degradation

To better understand the changes in the dynamics of p120-ctn subfamily proteins and E-cad at AJs, we performed immunofluorescence analysis of *L. interrogans*-infected RPTECs at 12 h and 24 h pi. Quantification of fluorescence intensity at cell-cell junctions showed that p0071, p120-ctn, and E-cad intensities significantly decreased at 12 h p.i. (*p <*0.01 for p0071, and *p <*0.05 for p120-ctn and E-cad), and these proteins were displaced continuously from the AJs (**Figures S3A–C**). We hypothesized that *L. interrogans* infection of RPTECs induces stressful stimuli that enhance E-cad endocytosis and p0071 and p120-ctn degradation through the lysosomal pathway and/or ubiquitinin-proteasomal system (UPS) (Flores-Maldonado et al., 2017). We then analyzed whether p0071 displaced from the AJs was associated with the endo-lysosomal pathway using a dual immunofluorescence staining to detect p0071 and the LAMP-1. AJ-disassociated p0071 were found in LAMP-1-positive-vacuoles, cytoplasmic LAMP-1-negative compartments, and nuclear compartments (**Figure S4A**).

Next, we analyzed the effect of pre-treating RPTECs with the lysosomal inhibitors [bafilomycin A (BF) or chloroquine (CQ)] or the proteasomal inhibitors [MG-132 (MG) or bortezomib (BZ)] before infection. All inhibitors did not significantly prevent degradation of p120-ctn family proteins (*p >*0.05) (**Figures S4B, C**). Notably, fragments reacting in p120-ctn immunoblots (between ~58 kDa and ~88 kDa) were detected when the proteasome was inhibited, suggesting that these fragments are p120-ctn-derived and may be targets of the UPS degradation pathway (**Figure S4B**). The immunoblots also suggested that limited proteolysis may occur upstream of the UPS since these putative UPS targets were smaller than the full-length (FL) protein. TEER decrease during *L. interrogans* infection of RPTECs (~20% of initial TEER) was prevented with the proteasomal inhibitors (~140% or ~95% of initial TEER with MG and BZ, respectively), but not with the lysosomal inhibitors (**Figure S4D**).

**Figure 5:**
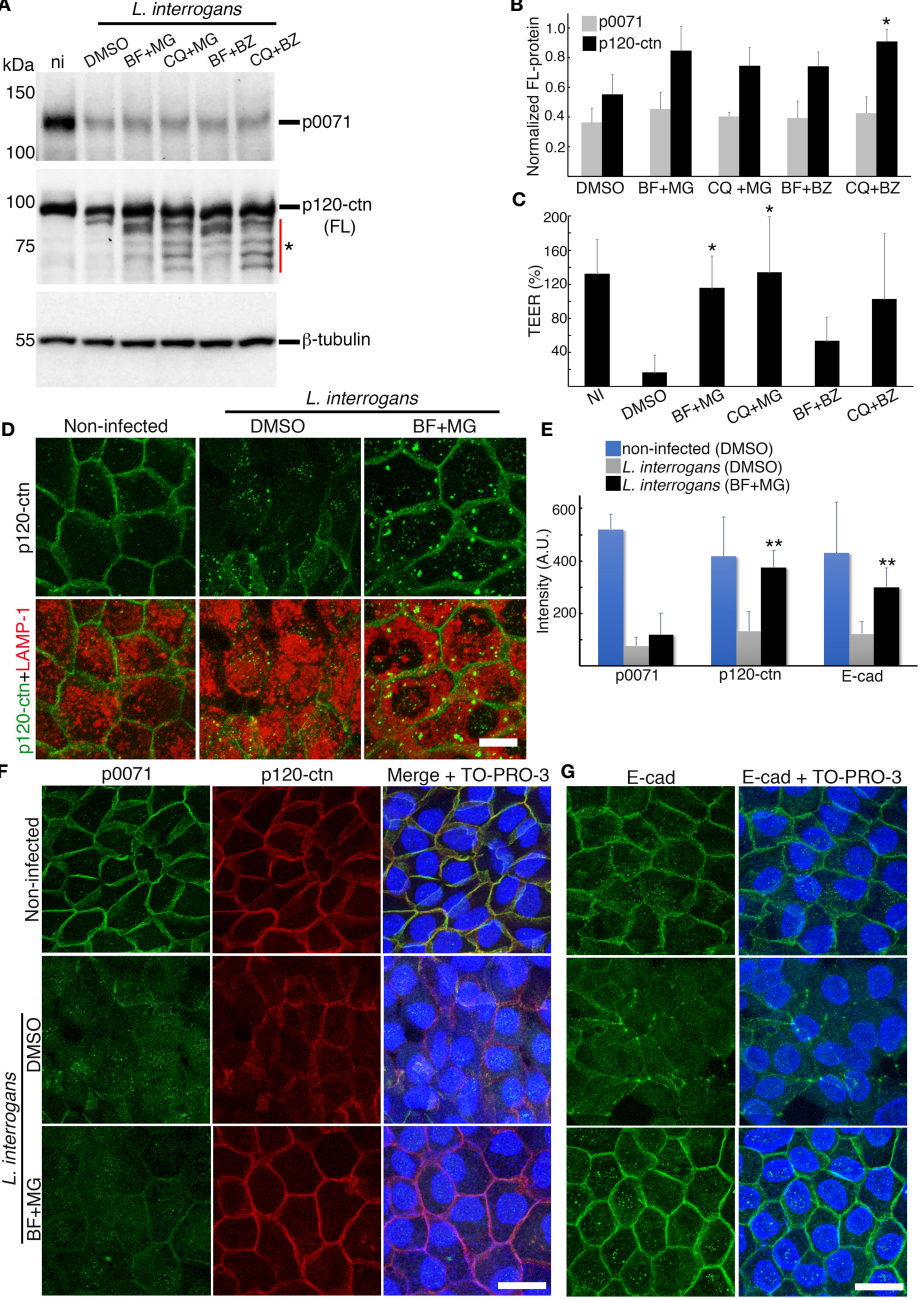
A lysosomal and proteasomal inhibitor combination prevents p120-ctn degradation. RPTECs were pre-treated for 30 min with a combination of lysosomal and proteasomal inhibitors and infected with *L. interrogans* (lysosomal inhibitors (bafilomycin A1 (BF) or chloroquine (CQ)) or the proteasomal inhibitors (MG-132 (MG) or bortezomib (BZ)). **(A)** Whole-cell lysates were subjected to western blotting with the indicated antibodies (18 h p.i.). Fragments that were newly detected by adding inhibitors are denoted by an asterisk. **(B)** Normalized levels of full-length (FL)-proteins. **(C)** Transepithelial electrical resistance (TEER) measurements at 18 h p.i. **(D)** RPTECs were pre-treated for 30 min with DMSO or BF+MG and infected with *L. interrogans*, fixed with 2% paraformaldehyde at 18 h p.i, and processed for immunostaining. p120-ctn was stained with an Alexa Fluor 488-labeled antibody (green) and LAMP-1 with a Cy3-labeled antibody (red) (see also **Figure S5**). **(E)** Quantification of fluorescence intensity at cell-cell junctions in RPTECs that were immunostained as described in **(F, G)**. A.U., arbitrary units. **(F, G)** Representative confocal images for non-infected and infected RPTECs. Cells were fixed with methanol at 18 h p.i. and processed for immunostaining. **(F)** p0071 was stained with an Alexa Fluor 488-labeled antibody (green) and p120-ctn with a Cy3-labeled antibody (red). **(G)** E-cad was stained with an Alexa Fluor 488-labeled antibody (green).The cell nuclei were stained with TO-PRO-3 (blue). Scale bar: 25 μm. Each bar represents the mean ± standard deviation of three independent experiments. *p <0.05 and **p <0.01.

Compensation mechanisms between degradative pathways have been reported because other systems must clear cellular materials that accumulate after inhibiting one degradative system to maintain homeostasis (Kocaturk and Gozuacik, 2018). Thus, we hypothesized that some ubiquitinated proteins accumulated after adding MG and BZ (**Figure S4E**) may be translocated to the lysosomal pathway for degradation. We then analyzed whether combining lysosomal and proteasomal inhibitors can prevent p0071 and p120-ctn degradation and found that such combination did not significantly prevent p0071 degradation (*p >*0.05) (**Figures 5A, B**). In contrast, FL-p120-ctn degradation tended to be suppressed by combining inhibitors and was significantly prevented by the combination of CQ+BZ (*p <*0.05) (**Figures 5A, B**). The *L. interrogans*-induced TEER decrease at 18 h p.i. (~17% of initial TEER) was prevented in infected RPTECs pretreated with a combination of BF+MG (~115% of initial TEER) and CQ+MG (~134% of initial TEER) (**Figure 5C**). The effects of BZ combined with either BF or CQ were not additive in terms of inhibiting the TEER decrease. Therefore, we used the BF+MG combination for further analysis using two different immunofluorescence protocols to detect total p120-ctn (**Figure 5D**) or AJ-associated p120-ctn (**Figures 5E, F**). Confocal images and quantification of fluorescence intensity at cell-cell junctions showed that p120-ctn accumulated in LAMP-1-positive vacuoles and also localized at AJs in BF+MG-treated RPTECs (**Figures 5D**, **S5**). The combination of BF+MG significantly prevented p120-ctn and E-cad displacement from the AJs (*p <*0.01) but not the displacement of p0071 (**Figures 5E–G**). The prevention of AJ disassembly without preventing p0071 degradation and displacement from AJs shows the redundant role of p0071 and p120-ctn as the AJ scaffold proteins. These results suggested that additional factors are involved in p0071 proteolysis.

### 3.5 Inhibiting proteasomal and lysosomal degradation prevents *L. interrogans*-induced RPTEC monolayer destruction

**Figure 6:**
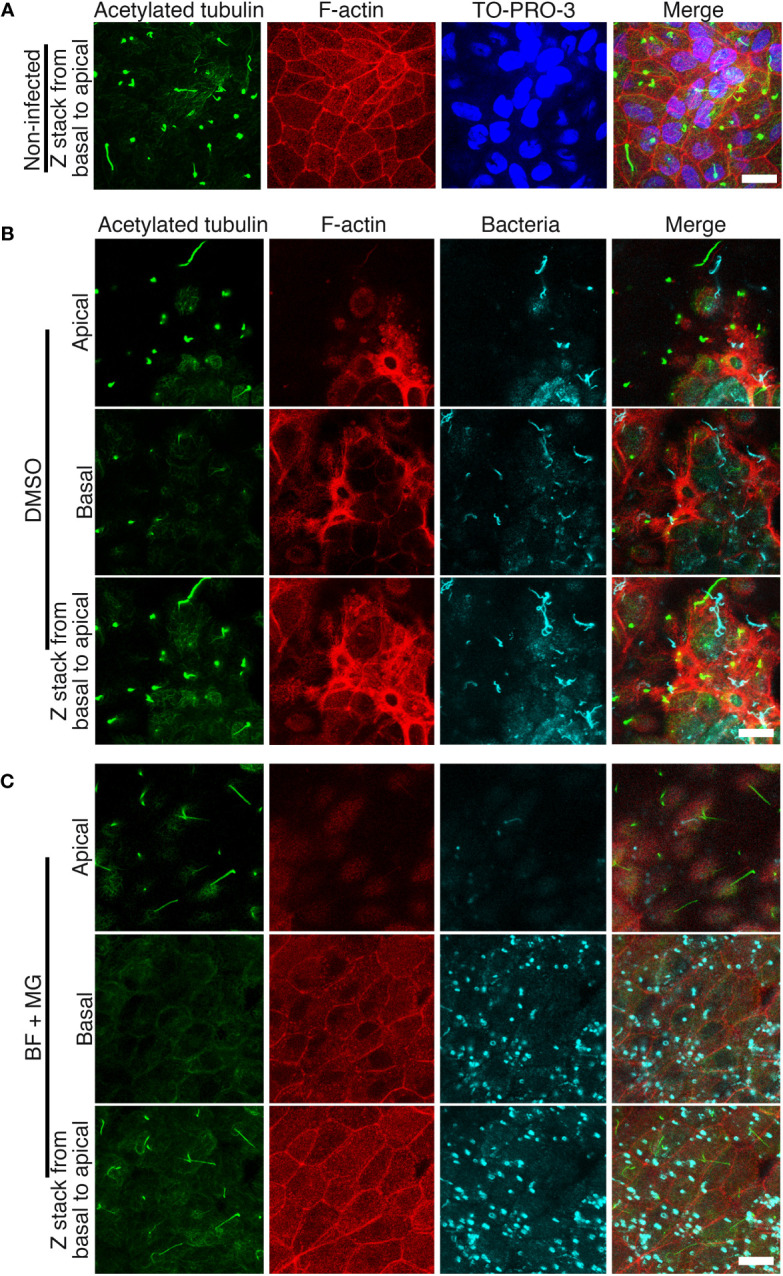
Inhibition of proteasomal and lysosomal degradation prevents the F-actin rearrangement induced by *L. interrogans.* Representative confocal images of **(A)** non-infected RPTECs or **(B, C)** *L. interrogans*-infected RPTECs. **(B)** RPTECs were pre-treated for 30 min with DMSO or **(C)** a combination of BF and MG. Infected RPTECs were fixed at 18 h p.i. and processed for immunostaining. Acetylated tubulin was stained with an Alexa Fluor 488-labeled antibody (green) and *L. interrogans* was stained with an Alexa 647-labeled antibody (cyan). F-actin was stained with rhodamine-phalloidin (red). The cell nuclei were stained with TO-PRO-3 (blue) in **(A)**. The apical, basal, and compiled Z-stack images from basal to apical are shown. Scale bar: 10 μm.

Some bacterial pathogens, such as *Helicobacter pylori* (Hatakeyama and Brzozowski, 2006), can induce AJ disassembly by altering epithelial cell polarization. Therefore, we tested the possibility that *L. interrogans* also affects RPTEC polarization. Acetylated tubulin, which is a marker of polarized epithelial cells, and F-actin organization in non-infected RPTECs (**Figure 6A**) were compared with RPTECs infected with *L. interrogans* in the absence or presence of BF+MG. *L. interrogans* induced F-actin rearrangement; however, acetylated tubulin was not affected (**Figure 6B**). This cytoskeletal disorganization was prevented by BF+MG inhibitors (**Figure 6C**). Leptospires were observed at the apical side in DMSO-treated RPTECs, while they were observed as dots near the basal side in BF+MG-treated RPTECs (**Figures 6B, C**).

**Figure 7:**
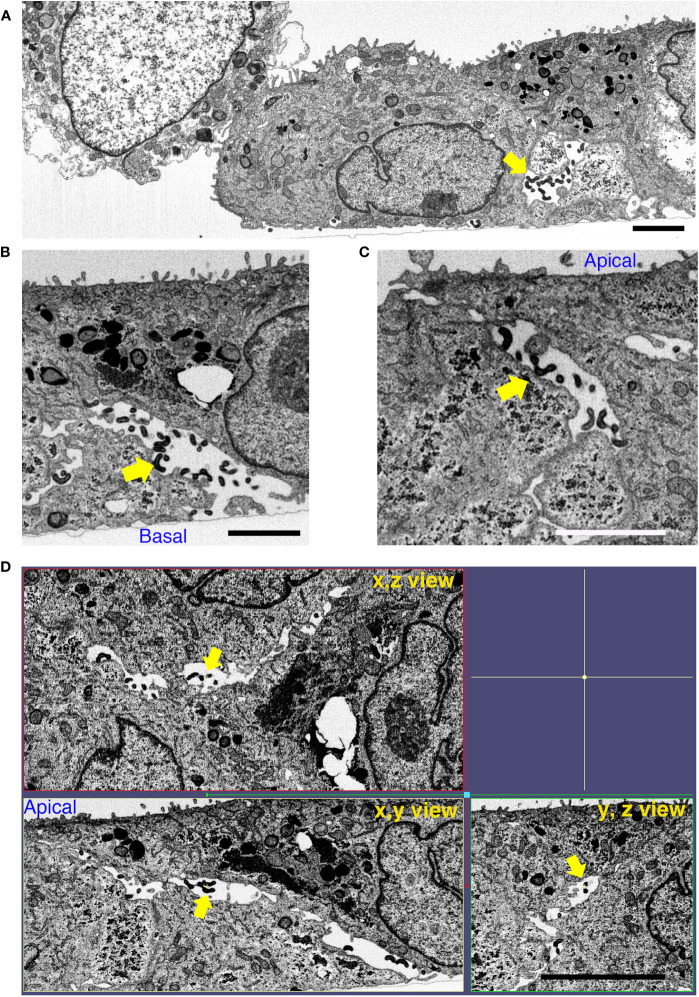
*L. interrogans* induces RPTEC monolayer destruction. RPTECs were pre-treated for 30 min with DMSO and infected with *L. interrogans*. Representative FIB-SEM images at 16 h p.i. **(A)** *L. interrogans* induces RPTEC monolayer destruction with apoptotic cells within the adhesion-compromised cells. **(B, C)** Leptospires transmigrate paracellularly from the basal to the apical side (yellow arrows). **(D)** The xyz view tomograms show the presence of large gaps between the cells, which are filled with paracellular leptospires (yellow arrows). Scale bars: 2 μm for **(A–C)** and 5 μm for **(D)**.

**Figure 8:**
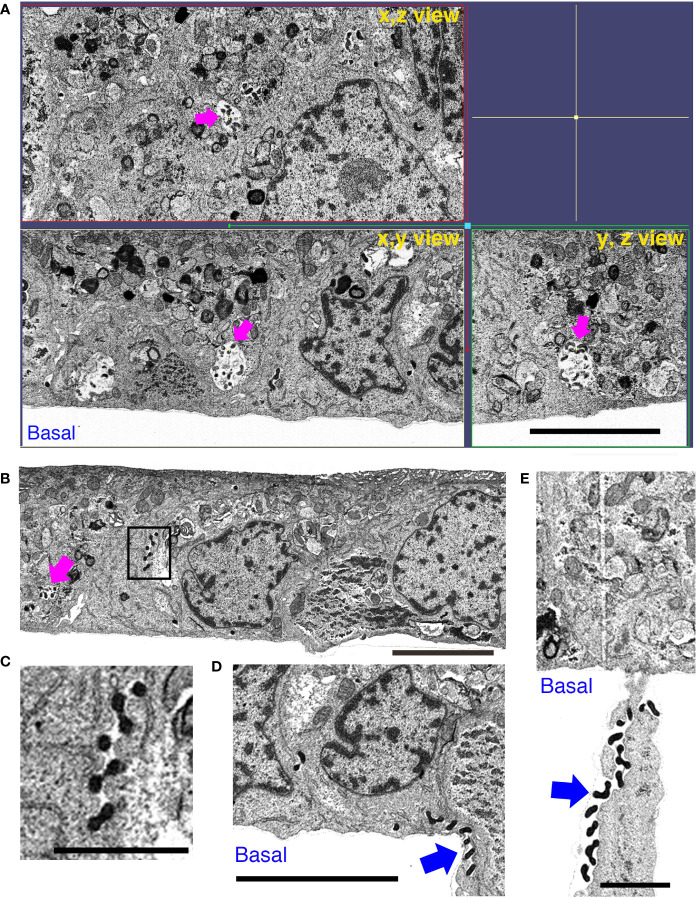
Inhibition of proteasomal and lysosomal degradation prevents the RPTEC monolayer destruction induced by *L. interrogans.* RPTECs were pre-treated for 30 min with BF+MG and infected with *L. interrogans.* Representative FIB-SEM images at 16 h p.i. **(A)** The xyz view tomograms show the absence of intercellular gaps and the presence of leptospires within intracellular vacuoles (magenta arrows). **(B)** Leptospires were observed within vacuoles (magenta arrow) or in tightly closed paracellular space (inset). **(C)** An enlargement of the inset in **(B)**. **(D, E)** Leptospires pass through the filter pore of the culture insert (blue arrows). Scale bars: 5 μm for **(A, B, D)** and 2 μm for **(C, E)**.

We performed focused ion beam-scanning electron microscopy (FIB/SEM) analysis, which has high image and spatial resolution, to further understand the effect of BF+MG during *L. interrogans* infection of RPTECs. AJ disruption by *L. interrogans* in the absence of inhibitors induced the appearance of large gaps within the cell monolayer. These intercellular spaces were filled with leptospires which were observed from the basal to the apical side (**Figures 7A–D** and **Video S1**). Some adhesion-compromised RPTECs were apoptotic which might represent cells that extrude from the monolayer (**Figure 7A**) (Grieve and Rabouille, 2014). The x, y, and z view of infected RPTECs showed the large spaces between the cells, which were absent in the BF+MG-treated RPTECs (**Figures 7D**, **8A**). In contrast, in BF+MG-treated cells, a continuous tight cell monolayer was observed (**Figure 8B** and **Video S2**). Leptospires were located proximally to the basal side within intracellular compartments that were found to be LAMP-1-positive by immunofluorescence (**Figures 8A**, **S6**) or were located extracellularly in a tightly controlled intercellular space (**Figures 8B, C**). Leptospires transmigrating from the basal side through the pore of the culture insert membrane which was filled with RPTEC-cytoplasm were also observed (**Figures 8D, E** and **Video S2**). These results indicate that *L. interrogans* does not affect epithelial cell polarization, and BF+MG inhibitors can prevent the AJ disassembly, F-actin rearrangement, and monolayer destruction induced by *L. interrogans*.

### 3.6 Z-VAD-FMK inhibits *L. interrogans*-dependent p0071 degradation and displacement of p120-ctn subfamily proteins from the AJ

**Figure 9:**
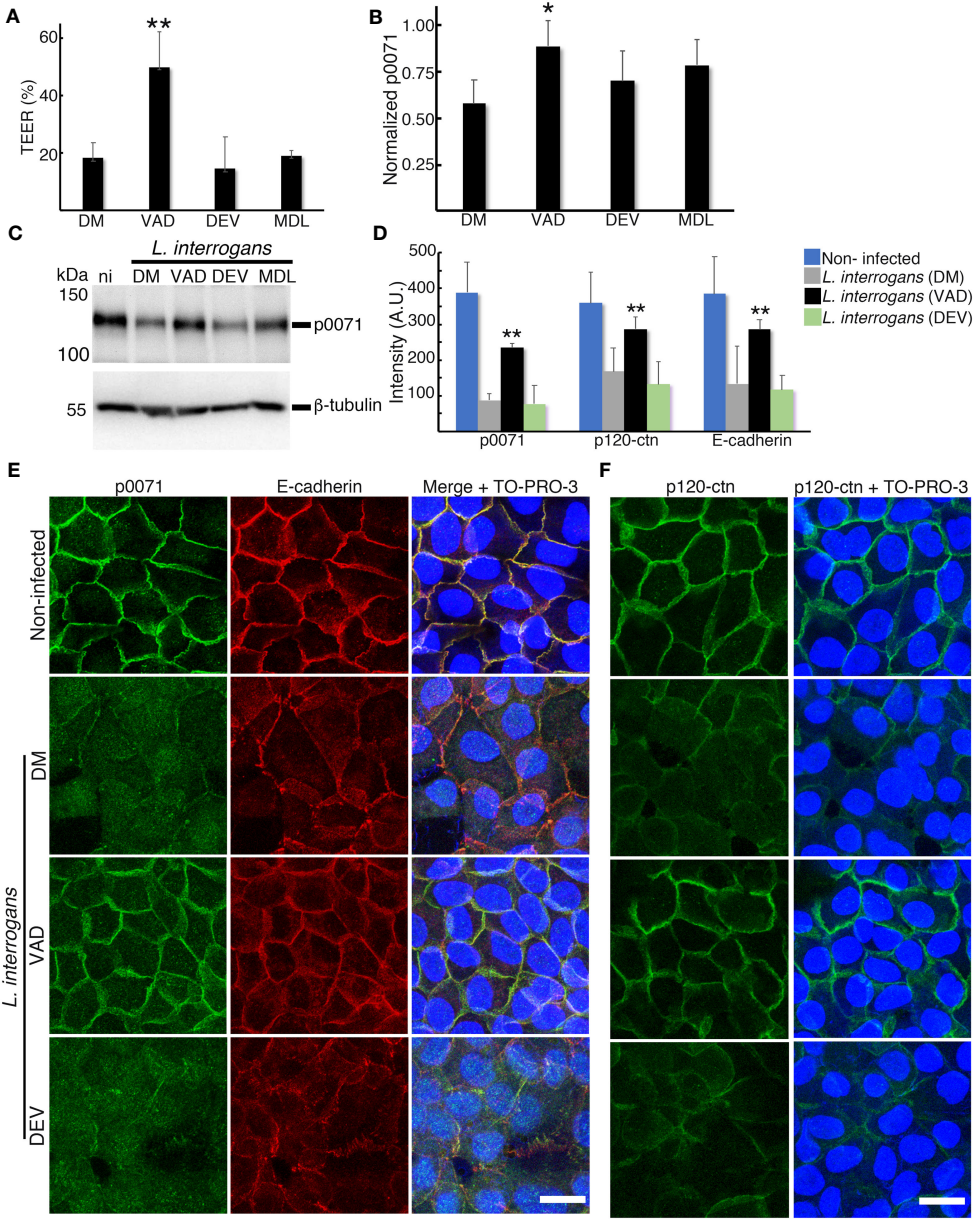
Z-VAD-FMK inhibits *L. interrogans*-dependent p0071 degradation and displacement of p120-ctn subfamily proteins from the AJs. RPTECs were pre-treated for 30 min with DMSO (DM), Z-VAD-FMK (VAD), Z-DEVD-FMK (DEV), or MDL-28170 (MDL), and infected with *L. interrogans*. **(A)** Transepithelial electrical resistance (TEER) measurements at 18 h p.i. **(B, C)** Whole-cell lysates were subjected to western blotting with the indicated antibodies (18 h p.i.). **(B)** Normalized levels of p0071. **(C)** Representative immunoblots. **(D)** Quantification of the fluorescence intensity at cell-cell junctions. A.U., arbitrary units. **(E, F)** Representative confocal images for infected RPTECs at 18 h p.i. **(E)** p0071 was stained with an Alexa Fluor 488-labeled antibody (green), and E-cad was stained with a Cy3-labeled antibody (red). **(F)** p120-ctn was stained with an Alexa Fluor 488-labeled antibody (green). The cell nuclei were stained with TO-PRO-3 (blue). Scale bar: 20 μm. Each bar represents the mean ± standard deviation of three independent experiments. *p <0.05 and **p <0.01.

**Figure 10:**
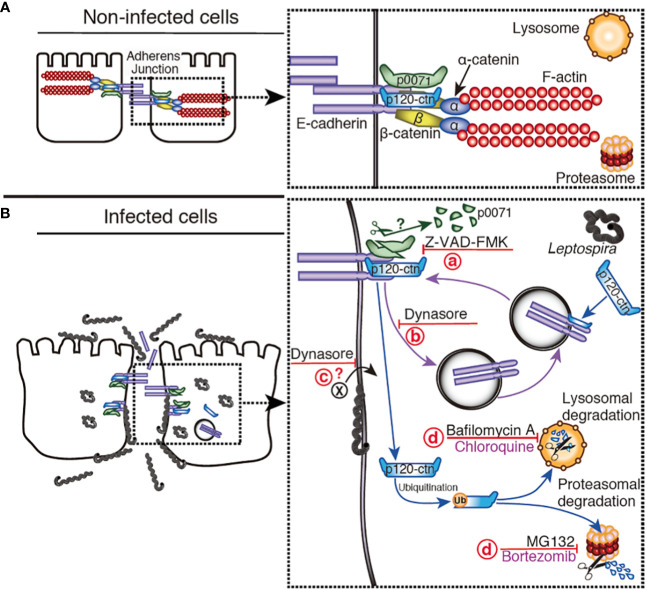
Hypothetical model representing the disassembly of AJs by *L. interrogans*. **(A)** Non-infected cells: E-cad is located at the plasma membrane stabilized by p0071 and p120-ctn, the cytoskeleton (F-actin) is linked to E-cad by α- and β-catenin, and the AJs are not disturbed. For clarity, the α/β-catenin complex and F-actin were omitted in the diagram of infected cells. **(B)** *L. interrogans*-infected cells: Leptospires are attached to the basolateral membrane, intracellularly located, or translocated to the apical side. (a) A protease inhibited by Z-VAD-FMK cleaves p0071, and induces p0071 and p120-ctn displacement from the AJs. (b) AJC complex endocytosis, which is inhibited by dynasore, may be enhanced. (c) The endocytosis of a leptospiral virulence factor may also be inhibited by dynasore. (d) p120-ctn degradation and monolayer integrity destruction can be inhibited by a combination of lysosomal and proteasomal inhibitors.

To test the possibility that p0071 is undergoing limited proteolysis during *L. interrogans* infection, we evaluated the effect of pre-treating RPTECs with the pan-caspase inhibitor Z-VAD-FMK and the calpain inhibitor MDL28170 before infection. The TEER decrease during *L. interrogans* RPTEC infection (~20% of initial TEER) was partially prevented by Z-VAD-FMK (~50% of initial TEER), but not by MDL28170 (~20% of initial TEER) (**Figure 9A**). Z-VAD-FMK significantly prevented p0071 degradation (*p <*0.05) (**Figures 9B, C**), displacement of p120-ctn subfamily proteins and E-cad from the AJs (**Figures 9D–F**), and partially prevented the F-actin rearrangement induced by *L. interrogans* (**Figure S7**), Since *L. interrogans* induces a caspase-3-dependent cell death in renal epithelial cells in animal models (Marinho et al., 2015), the effects of pre-treating RPTECs before infection with Z-DEVD-FMK were also evaluated to rule out the possibility that the inhibitory effects of Z-VAD-FMK are caspase-3-dependent events. No inhibitory effects were detected in *L. interrogans*-induced TEER decrease, p0071 degradation, and AJ-proteins mislocalization (**Figures 9A–F**). These results suggested that proteolysis by a protease inhibited by Z-VAD-FMK is involved in p0071 degradation and displacement of p120-ctn subfamily proteins from the AJs (**Figure 10**).

## 4 Discussion

AJs are the primary target during AJC disassembly induced by *L. interrogans* in epithelial and endothelial cells (Sato and Coburn, 2017; Sebastián et al, 2021). AJs can be disturbed from the outside as a consequence of E-cad proteolysis by bacterial proteases or eukaryotic proteases activation that cleave the E-cad extracellular domains (Hoy et al., 2010; Backert et al., 2018; Devaux et al., 2019; Hsu et al., 2021). Altering AJ-proteins from the inside can also be achieved by proteases, activation of degradation pathways, or post-translational modifications (Huber, 2020). In this study, we demonstrate that *L. interrogans* disturb the AJs from the inside by specifically inducing p0071 and p120-ctn degradation, which are multifunctional E-cad- stabilizing proteins with armadillo domains. Human ctns containing armadillo domains that mediate cell-cell adhesion are divided into three subfamilies: p120-ctn subfamily, plakophilin subfamily (plakophilins 1–3), and β-catenin subfamily (β-ctn and plakoglobin) (Keil et al., 2013). p120-ctn is the prototypic and the most studied member of the p120-ctn subfamily, while p0071 is a relatively understudied member of this subfamily. p120-ctn is abundant in most epithelial cell lines, and its close relatives are poorly expressed; however, our data indicated that p0071 is also abundant in RPTECs. Depending on their relative abundance, members of the p120-ctn subfamily can functionally substitute for p120-ctn (Davis et al., 2003). Structurally, the p120-ctn proteins share a central domain comprising nine armadillo repeat motifs, whereas p0071 carries a PDZ-binding motif at its carboxyl terminus, which is absent in p120-ctn (Keil et al., 2013). Most characterized binding partners of p0071 interact with its armadillo repeat region or the PDZ-binding site; thus, p0071-specific functions can be disturbed during leptospiral pathogenesis. Besides the p0071 binding to proteins involved in cell adhesion, putative binding partners include Rab11 (Keil et al., 2013). A Rab11- dependent pathway is involved in recycling E-cad to the plasma membrane (Keil and Hatzfeld, 2014); therefore, the decrease in p0071 during *L. interrogans* infection may have deleterious effects on both AJC disassembly and recovery after a stressful stimulus. Additional effects induced by the decreased p120-ctn subfamily protein level include the dysregulation of Rho family GTPases that are regulated by p120-ctn, which can promote cytoskeletal rearrangement and synergistically act to disassemble the AJC (Sebastián et al., 2021).

The physically binding of p0071 and p120-ctn scaffold proteins to E-cad JMD masks the endocytic signal and stabilizes E-cad at the plasma membrane (Rusu and Georgiou, 2020). The constitutive turnover of E-cad in cell monolayers primarily results from active internalization and recycling processes. Active E-cad internalization displaces scaffold proteins from the AJs, which can be recycled back to the plasma membrane and fuels AJ dynamics and the remodeling necessary for epithelial tissues homeostasis (Katsuno-Kambe and Yap, 2020). Various stimuli, including cytokines, bacterial adherence, toxins, and oxidative stress can increase the E-cad endocytosis rate through pathological signals (Utech et al., 2010). *L. interrogans* induces the degradation of both proteins as an efficient strategy to increase E-cad endocytosis and inhibit E-cad recycling and transfer to the plasma membrane, thus, disassembling the AJs. The identification of the pathological signal that triggers E-cad endocytosis was not the focus of this study; however, our results show that the elucidation of this signal is crucial for preventing AJ disruption and controlling bacterial dissemination in the host.

Clathrin-mediated-endocytosis triggers AJ disassembly. Although we have only considered the inhibition of E-cad endocytosis, we could not rule out the possibility that dynasore inhibited multiple pathways, i.e., inhibition of E-cad endocytosis and a clathrin-mediated leptospiral toxin-uptake. Bacterial toxins, including diphtheria toxin and *Mycoplasma pneumoniae* community-acquired respiratory distress syndrome toxin, are endocytosed through clathrin-dependent endocytosis (Krishnan et al., 2013). In pathogenic *Leptospira*, virulence-modifying proteins (LA3490, LA0620, LA1402, and LA0591) encoded by the PF07589 gene family are AB toxins with a putative mechanism of cellular entry similar to the community-acquired respiratory distress syndrome toxin. Virulence-modifying proteins have contributed to leptospirosis pathogenesis through cytoskeleton disassembly, DNA degradation, and caspase-3 activation (Chaurasia and Vinetz, 2022). Using microarray transcriptional analysis, our research group previously found that LA1402 and LA0620 were upregulated in the virulent *L. interrogans* strain when compared with its culture-attenuate derivative strain (Toma et al., 2014). Therefore, the role of these virulence-modifying proteins in AJ disassembly warrants further investigation.

Proteasomal inhibitors can prevent the *L. interrogans*-induced TEER decrease. Thus, this is the first study to show that *L. interrogans* hijacks the eukaryotic UPS for its benefit. Protein degradation by the UPS requires the addition of ubiquitin at specific lysines, and p120-ctn possesses several lysines that can be ubiquitinated (Keil et al., 2013). However, we were unable to detect p120-ctn ubiquitination or knockdown p120-ctn expression to strengthen our results, which are limitations of our study. The UPS may also be involved in the degradation of additional factors that stabilizes cell-cell junctions. Some candidates are the Rho GTPases including Rac1, Cdc42, and RhoA proteins, which are essential regulatory molecules that control actin cytoskeleton organization and dynamic. Rac1 was identified with the highest degree value in a bioinformatic analysis that identifies *Leptospira* host-interacting proteins (Kumar et al., 2019).

Limited proteolysis plays a major role in the selective degradation of proteins from oligomeric complexes in which the generated fragments may transiently reside, thus perturbing the proper biological function of the complexes (Piatkov et al., 2014). Caspases and calpains are the host cysteine proteases involved in this limited proteolysis of target proteins via cleavage of polypeptide chains at specific sites that generates protein fragments with destabilizing Nt residues; therefore, they are targeted for degradation by the N-end rule pathway (Minina et al., 2017). The N-end rule pathway may have a dual role in regulating bulk degradation by lysosomes and selective protein degradation through the UPS (Gibbs et al., 2014). Limited proteolysis and digestive proteolysis acting in tandem may represent a powerful mechanism to modify proteomes during bacterial infection. Our results are compatible with a hypothetical model for *L. interrogans*-induced degradation of p0071 via a protease inhibited by Z-VAD-FMK which inhibition prevented the disassembly of the AJ (**Figure 10**). Z-VAD-FMK is a cysteine protease inhibitor that inhibits eukaryotic caspases which is a family of 12 proteases (caspases 1–12) (Poreba et al., 2013). However, we could not rule out the possibility that this inhibitor inhibits a bacterial protease since chlamydial protease is known to be inhibited by Z-WEHD-FMK, an inhibitor of eukaryotic caspase-1 (Christian et al., 2011). Furthermore, several caspase-like bacterial peptidases have been identified (Johnson et al., 2022). Leptospiral proteases are essential for host-pathogen interaction because they are differentially expressed and upregulated in response to the physiological temperature and osmolarity (Dhandapani et al., 2018). Biochemical characterization of leptospiral proteases showed that they degrade extracellular matrix and plasma proteins, but protease-degrading AJ proteins have not been reported (da Silva et al., 2018). Bioinformatics analysis has identified 104 proteases, including cysteine proteases, whose role in pathogenesis remains unknown (Dhandapani et al., 2018). Future studies are needed to clarify if some of these proteases are involved in AJ disassembly.

In conclusion, our study showed that *L. interrogans* targets p0071 and p120-ctn for degradation, which are two critical players in the regulation of AJ stability. Our results show that inhibition of the two major eukaryotic protein degradation pathways can prevent AJ disassembly and monolayer destruction which has novel relevance in understanding the mechanisms of leptospirosis pathogenesis. This study is the first to provide evidence that *L. interrogans* hijacks the UPS to achieve successful infection. The identification of the bacterial factors involved in this critical pathogenic step will provide a foundation for the development of candidate vaccine and diagnostic antigens.

## Data availability statement

The datasets presented in this study can be found in online repositories. The names of the repository/repositories and accession number(s) can be found in the article/**Supplementary Material**.

## Ethics statement

Ethical approval was not required for the studies on humans in accordance with the local legislation and institutional requirements because only commercially available established cell lines were used.

## Author contributions

Conception, administration and supervision of the project: CT. Investigation: RT, IS, and CT. Methodology: RT, IS, BH, NO, HY, and CT. Data analysis, RT, IS, BH, HY, TY, and CT. Resources: TY. Funding acquisition: BH and CT. CT wrote the original draft. All authors contributed to the article and approved the submitted version.

## Supplementary material

Supplementary Figure 1
*L. interrogans* induces aberrant p0071 localization and partial plakoglobin mislocalization. **(A)** Representative confocal images of *L. biflexa* or *L. interrogans*-infected RPTECs at 24 h p.i. p0071 was stained with an Alexa Fluor 488-labeled antibody (green), and plakoglobin was stained with a Cy3-labeled antibody (red). The cell nuclei and leptospiral DNA were counterstained with TO-PRO-3 (cyan). Scale bar: 10 μm. **(B)** Quantification of fluorescence intensity at cell-cell junctions. Each bar represents the mean ± standard deviation of three independent experiments. A.U.: arbitrary units. ***p <*0.01.

Supplementary Figure 2 Dynasore does not affect p0071 and p120-ctn localization in non-infected RPTECs. DMSO- or Dynasore-pre-treated RPTECs were fixed and processed for immunofluorescence analysis. RPTECs were immunostained with anti-p0071 **(A)** or p120-ctn **(B)** antibodies and then visualized with Alexa Fluor 488-conjugated secondary antibodies (green). E-cad was detected using a Cy3-labeled antibody (red) and the cell nuclei were detected with TO-PRO-3 (blue). Scale bar: 10 μm.

Supplementary Figure 3 Dynamics of E-cad, p0071, and p120-ctn localization at AJs during *L. interrogans* infection of RPTECs. **(A, B)** Representative confocal images of non-infected or *L. interrogans*-infected RPTECs. Cells were methanol fixed and processed for immunostaining at 12 h and 24 h p.i. **(A)** p0071 was stained with an Alexa Fluor 488-labeled antibody (green), and p120-ctn was stained with a Cy3-labeled antibody (red). **(B)** E-cad was stained with an Cy3-labeled antibody (red). **(A, B)** The cell nuclei were counterstained with TO-PRO-3 (blue). Scale bar: 10 μm. **(C)** Quantification of fluorescence intensity at cell-cell junctions. Each bar represents the mean ± standard deviation of three independent experiments. A.U.: arbitrary units. **p <*0.05 and ***p <*0.01.

Supplementary Figure 4 Lysosomal and proteasomal inhibitors are unable to prevent p0071 and p120-ctn degradation. **(A)** Dual immunofluorescence staining to detect p0071 (green) and the lysosomal associated membrane protein (LAMP-1, red) in non-infected and *L. interrogans*-infected RPTECs at 24 h p.i. Scale bar: 5 μm. **(B)** RPTECs were pre-treated for 30 min with either the lysosomal inhibitors (bafilomycin A1 (BF) or chloroquine (CQ)) or the proteasomal inhibitors (MG-132 (MG) or bortezomib (BZ)) and infected with *L. interrogans.* Whole-cell lysates were subjected to western blotting at 18 h p.i. Fragments that were newly detected by adding inhibitors are denoted by an asterisk. **(C)** Normalized levels of full-length (FL)-proteins. Each bar represents the mean ± standard deviation of three independent experiments. **(D)** Transepithelial electrical resistance (TEER) measurements at 18 h p.i., ***p <*0.01. **(E)** Detection of total ubiquitinated-proteins in *L. interrogans*-infected RPTECs after the addition of the inhibitors as described in **(B)**.

Supplementary Figure 5 p120-ctn localization in LAMP-1-positive vacuoles. RPTECs were pre-treated for 30 min with DMSO, dynasore, MG, or BF+MG; infected with *L. interrogans* for 18 h; and processed for immunofluorescence. p120-ctn was stained with an Alexa Fluor 488-labeled antibody (green), while LAMP-1 was stained with a Cy3-labeled antibody (red). The cell nuclei were stained with TO-PRO-3 (blue). Scale bar: 20 μm.

Supplementary Figure 6
*L. interrogans* localizes in LAMP-1-positive vacuoles. RPTECs were pre-treated with DMSO or BF+MG and infected with *L. interrogans*. Representative confocal images showing infected RPTECs (18 h p.i.). LAMP-1 was stained with an Alexa Fluor 488-labeled antibody (green), while *L. interrogans* was stained with a Cy3-labeled antibody (red). The cell nuclei were stained with TO-PRO-3 (blue). Scale bar: 10 μm.

Supplementary Figure 7 Z-VAD-FMK partially prevents *L. interrogans*-induced F-actin rearrangement. Representative confocal images of **(A)** non-infected RPTECs or **(B)** *L. interrogans*-infected RPTECs. **(B)** RPTECs were pre-treated for 30 min with DMSO, BF+MG, or Z-VAD-FMK and infected with *L. interrogans* for 24 h. *L. interrogans* was stained with an Alexa 647-labeled antibody (cyan) and F-actin with rhodamine-phalloidin (red). The cell nuclei were stained with TO-PRO-3 (blue) in **(A)**. Scale bars: 20 μm.

Supplementary Video 1
*L. interrogans* transmigrates through the paracellular route. FIB-SEM tomography of DMSO-treated RPTECs reveals extracellular leptospires in large intercellular spaces (related to **Figure 7**). Scale bar: 5 μm.

Supplementary Video 2 Inhibition of proteasomal and lysosomal degradation prevents the RPTEC monolayer destruction induced by *L. interrogans.* FIB-SEM tomography of BF+MG-treated RPTECs reveals leptospires in intracellular spaces (related to **Figure 8**). Scale bar: 5 μm.

## References

[B1] Ashida H. Sasakawa C. (2017). Bacterial E3 ligase effectors exploit host ubiquitin systems. Curr. Opin. Microbiol. 35, 16–22. doi: 10.1016/j.mib.2016.11.001 27907841

[B2] Backert S. Bernegger S. Skórko-Glonek J. Wessler S. (2018). Extracellular HtrA serine proteases: An emerging new strategy in bacterial pathogenesis. Cell. Microbiol. 20, e12845. doi: 10.1111/cmi.12845 29582532

[B3] Barocchi M. A. Ko A. I. Reis M. G. McDonald K. L. Riley L. W. (2002). Rapid translocation of polarized MDCK cell monolayers by *Leptospira interrogans*, an invasive but nonintracellular pathogen. Infect. Immun. 70, 6926–6932. doi: 10.1128/iai.70.12.6926-6932.2002 12438371 PMC132952

[B4] Bergounioux J. Elisee R. Prunier A. L. Donnadieu F. Sperandio B. Sansonetti P.. (2012). Calpain activation by the *Shigella flexneri* effector VirA regulates key steps in the formation and life of the bacterium's epithelial niche. Cell Host Microbe 11, 240–252. doi: 10.1016/j.chom.2012.01.013 22423964

[B5] Bierque E. Soupé-Gilbert M. E. Thibeaux R. Girault D. Guentas L. Goarant C. (2020). *Leptospira interrogans* retains direct virulence after long starvation in water. Curr. Microbiol. 77, 3035–3043. doi: 10.1007/s00284-020-02128-7 32683468

[B6] Cai J. Culley M. K. Zhao Y. Zhao J. (2018). The role of ubiquitination and deubiquitination in the regulation of cell junctions. Protein Cell 9, 754–769. doi: 10.1007/s13238-017-0486-3 29080116 PMC6107491

[B7] Chaurasia R. Vinetz J. M. (2022). In silico prediction of molecular mechanisms of toxicity mediated by the leptospiral PF07598 gene family-encoded virulence-modifying proteins. Front. Mol. Biosci. 9. doi: 10.3389/fmolb.2022.1092197 PMC9900628 36756251

[B8] Christian J. G. Heymann J. Paschen S. A. Vier J. Schauenburg L. Rupp J.. (2011). Targeting of a chlamydial protease impedes intracellular bacterial growth. PloS Pathog. 7, e1002283. doi: 10.1371/journal.ppat.1002283 21990969 PMC3182938

[B9] Coburn J. Picardeau M. Woods C. W. Veldman T. Haake D. A. (2021). Pathogenesis insights from an ancient and ubiquitous spirochete. PloS Pathog. 17, e1009836. doi: 10.1371/journal.ppat.1009836 34673833 PMC8530280

[B10] Courrol D. D. S. da Silva C. C. F. Prado L. G. Chura-Chambi R. M. Morganti L. de Souza G. O.. (2022). Leptolysin, a *Leptospira* secreted metalloprotease of the pappalysin family with broad-spectrum activity. Front. Cell. Infect. Microbiol. 12. doi: 10.3389/fcimb.2022.966370 PMC9445424 36081769

[B11] Daroz B. B. Fernandes L. G. V. Cavenague M. F. Kochi L. T. Passalia F. J. Takahashi M. B.. (2021). A review on host-*Leptospira* interactions: What we know and future expectations. Front. Cell. Infect. Microbiol. 11. doi: 10.3389/fcimb.2021.777709 PMC8657130 34900757

[B12] Daroz B. B. Fernandes L. G. V. Teixeira A. F. Nascimento A. L. T. O. (2020). In silico structural and functional characterization of HtrA proteins of *Leptospira* spp.: Possible implications in pathogenesis. Trop. Med. Infect. Dis. 5, 179. doi: 10.3390/tropicalmed5040179 33260771 PMC7709667

[B13] da Silva L. B. Menezes M. C. Kitano E. S. Oliveira A. K. Abreu A. G. Souza G. O.. (2018). *Leptospira interrogans* secreted proteases degrade extracellular matrix and plasma proteins from the host. Front. Cell. Infect. Microbiol. 8. doi: 10.3389/fcimb.2018.00092 PMC5881292 29637048

[B14] Davis M. A. Ireton R. C. Reynolds A. B. (2003). A core function for p120-catenin in cadherin turnover. J. Cell. Biol. 163, 525–534. doi: 10.1083/jcb.200307111 14610055 PMC2173649

[B15] Devaux C. A. Mezouar S. Mege J. L. (2019). The E-cadherin cleavage associated to pathogenic bacteria infections can favor bacterial invasion and transmigration, dysregulation of the immune response and cancer induction in humans. Front. Microbiol. 10. doi: 10.3389/fmicb.2019.02598 PMC6857109 31781079

[B16] Dhandapani G. Sikha T. Pinto S. M. KIran Kumar M. K. Patel K. Kumar M.. (2018). Proteomic approach and expression analysis revealed the differential expression of predicted leptospiral proteases capable of ECM degradation. Biochim. Biophy.s Acta Proteins Proteom. 1866, 712–721. doi: 10.1016/j.bbapap.2018.04.006 29654978

[B17] Díaz-Díaz C. Baonza G. Martín-Belmonte F. (2020). The vertebrate epithelial apical junctional complex: Dynamic interplay between Rho GTPase activity and cell polarization processes. Biochim. Biophy.s Acta Biomembr. 1862, 183398. doi: 10.1016/j.bbamem.2020.183398 32561145

[B18] Fiskin E. Bionda T. Dikic I. Behrends C. (2016). Global analysis of host and bacterial ubiquitinome in response to *Salmonella* Typhimurium infection. Mol. Cell. 62, 967–981. doi: 10.1016/j.molcel.2016.04.015 27211868

[B19] Flores-Maldonado C. Verdejo-Torres O. Campos-Blázquez J. Cabrera A. R. García-Hernández V. Rincón-Heredia R.. (2017). “Lysosomal degradation of junctional proteins,” in Lysosomes - associated diseases and methods to study their function. Ed. Dhiman P. (Rijeka, Croatia: IntechOpen). doi: 10.5772/intechopen.69370

[B20] Gibbs D. J. Bacardit J. Bachmair A. Holdsworth M. J. (2014). The eukaryotic N-end rule pathway: Conserved mechanisms and diverse functions. Trends Cell. Biol. 24, 603–611. doi: 10.1016/j.tcb.2014.05.001 24874449

[B21] Grieve A. G. Rabouille C. (2014). Extracellular cleavage of E-cadherin promotes epithelial cell extrusion. J. Cell Sci. 127, 3331–3346. doi: 10.1242/jcs.147926 24895403

[B22] Hartsock A. Nelson W. J. (2012). Competitive regulation of E-cadherin juxtamembrane domain degradation by p120-catenin binding and Hakai-mediated ubiquitination. PloS One 7, e37476. doi: 10.1371/journal.pone.0037476 22693575 PMC3365061

[B23] Hatakeyama M. Brzozowski T. (2006). Pathogenesis of *Helicobacter pylori* infection. Helicobacter 11 (Suppl 1), 14–20. doi: 10.1111/j.1478-405X.2006.00424.x 16925606

[B24] Hofmann I. Schlechter T. Kuhn C. Hergt M. Franke W. W. (2009). Protein p0071 - An armadillo plaque protein that characterizes a specific subtype of adherens junctions. J. Cell Sci. 122, 21–24. doi: 10.1242/jcs.043927 19092057

[B25] Hoy B. Löwer M. Weydig C. Carra G. Tegtmeyer N. Geppert T.. (2010). *Helicobacter pylori* HtrA is a new secreted virulence factor that cleaves E-cadherin to disrupt intercellular adhesion. EMBO Rep. 11, 798–804. doi: 10.1038/embor.2010.114 20814423 PMC2948180

[B26] Hsu S. H. Chou L. F. Hong C. H. Chang M. Y. Tsai C. Y. Tian Y. C.. (2021). Crosstalk between E-cadherin/β-catenin and NF-κB signaling pathways: The regulation of host-pathogen interaction during leptospirosis. Int. J. Mol. Sci. 22, 13132. doi: 10.3390/ijms222313132 34884937 PMC8658460

[B27] Huber P. (2020). Targeting of the apical junctional complex by bacterial pathogens. Biochim. Biophys. Acta Biomembr. 1862, 183237. doi: 10.1016/j.bbamem.2020.183237 32126234

[B28] Inamasu Y. Nikaido Y. Miyahara S. Maruoka T. Takigawa T. Ogawa M.. (2022). Dissemination of *Leptospira* into the intestinal tract resulting in fecal excretion in a hamster model of subcutaneous infection with *Leptospira interrogans* . Microb. Pathog. 165, 105481. doi: 10.1016/j.micpath.2022.105481 35292370

[B29] Johnson A. G. Wein T. Mayer M. L. Duncan-Lowey B. Yirmiya E. Oppenheimer-Shaanan Y.. (2022). Bacterial gasdermins reveal an ancient mechanism of cell death. Science 375, 221–225. doi: 10.1126/science.abj8432 35025633 PMC9134750

[B30] Katsuno-Kambe H. Yap A. S. (2020). Endocytosis, cadherins and tissue dynamics. Traffic 21, 268–273. doi: 10.1111/tra.12721 31912628

[B31] Keil R. Hatzfeld M. (2014). The armadillo protein p0071 is involved in Rab11-dependent recycling. J. Cell. Sci. 127, 60–71. doi: 10.1242/jcs.132266 24163434

[B32] Keil R. Schulz J. Hatzfeld M. (2013). p0071/PKP4, a multifunctional protein coordinating cell adhesion with cytoskeletal organization. Biol. Chem. 394, 1005–1017. doi: 10.1515/hsz-2013-0114 23640939

[B33] Keller A. Nesvizhskii A. I. Kolker E. Aebersold R. (2002). Empirical statistical model to estimate the accuracy of peptide identifications made by MS/MS and database search. Anal. Chem. 74, 5383–5392. doi: 10.1021/ac025747h 12403597

[B34] Kizilyaprak C. Longo G. Daraspe J. Humbel B. M. (2015). Investigation of resins suitable for the preparation of biological sample for 3-D electron microscopy. J. Struct. Biol. 189, 135–146. doi: 10.1016/j.jsb.2014.10.009 25433274

[B35] Kizilyaprak C. Stierhof Y. D. Humbel B. M. (2019). Volume microscopy in biology: FIB-SEM tomography. Tissue Cell 57, 123–128. doi: 10.1016/j.tice.2018.09.006 30385054

[B36] Kocaturk N. M. Gozuacik D. (2018). Crosstalk between mamMalian autophagy and the ubiquitin-proteasome system. Front. Cell Dev. Biol. 6. doi: 10.3389/fcell.2018.00128 PMC6175981 30333975

[B37] Koizumi N. Watanabe H. (2004). Leptospiral immunoglobulin-like proteins elicit protective immunity. Vaccine 22, 1545–1552. doi: 10.1016/j.vaccine.2003.10.007 15063580

[B38] Kremer J. R. Mastronarde D. N. McIntosh J. R. (1996). Computer visualization of three-dimensional image data using IMOD. J. Struct. Biol. 116, 71–76. doi: 10.1006/jsbi.1996.0013 8742726

[B39] Krishnan M. Kannan T. R. Baseman J. B. (2013). *Mycoplasma pneumoniae* CARDS toxin is internalized via clathrin-mediated endocytosis. PloS One 8, e62706. doi: 10.1371/journal.pone.0062706 23667510 PMC3647021

[B40] Kumar S. Lata K. S. Sharma P. Bhairappanavar S. B. Soni S. Das J. (2019). Inferring pathogen-host interactions between *Leptospira interrogans* and *Homo sapiens* using network theory. Sci. Rep. 9, 1434. doi: 10.1038/s41598-018-38329-1 30723266 PMC6363727

[B41] Laemmli U. K. (1970). Cleavage of structural proteins during the assembly of the head of bacteriophage T4. Nature 227, 680–685. doi: 10.1038/227680a0 5432063

[B42] Mann M. Wilm M. (1994). Error-tolerant identification of peptides in sequence databases by peptide sequence tags. Anal. Chem. 66, 4390–4399. doi: 10.1021/ac00096a002 7847635

[B43] Marshall R. B. (1976). The route of entry of leptospires into the kidney tubule. J. Med. Microbiol. 9, 149–152. doi: 10.1099/00222615-9-2-149 933147

[B44] Medvetz D. A. Khabibullin D. Hariharan V. Ongusaha P. P. Goncharova E. A. Schlechter T.. (2012). Folliculin, the product of the Birt-Hogg-Dube tumor suppressor gene, interacts with the adherens junction protein p0071 to regulate cell-cell adhesion. PloS One 7, e47842. doi: 10.1371/journal.pone.0047842 23139756 PMC3490959

[B45] Minina E. A. Moschou P. N. Bozhkov P. V. (2017). Limited and digestive proteolysis: Crosstalk between evolutionary conserved pathways. New. Phytol. 215, 958–964. doi: 10.1111/nph.14627 28574164

[B46] Miyahara S. Saito M. Kanemaru T. Villanueva S. Y. Gloriani N. G. Yoshida S. (2014). Destruction of the hepatocyte junction by intercellular invasion of *Leptospira* causes jaundice in a hamster model of Weil's disease. Int. J. Exp. Pathol. 95, 271–281. doi: 10.1111/iep.12085 24945433 PMC4170969

[B47] Nikaido Y. Ogawa M. Fukuda K. Yokoyama M. Kanemaru T. Nakayama T.. (2019). Transbronchial invasion and proliferation of *Leptospira interrogans* in lung without inflammatory cell infiltration in a hamster model. Infect. Immun. 87, e00727–e00719. doi: 10.1128/iai.00727-19 PMC6867870 31548321

[B48] Okuda S. Watanabe Y. Moriya Y. Kawano S. Yamamoto T. Matsumoto M.. (2017). jPOSTrepo: An international standard data repository for proteomes. Nucleic Acids Res. 45, D1107–D1111. doi: 10.1093/nar/gkw1080 27899654 PMC5210561

[B49] Peng L. Kapp E. A. McLauchlan D. Jordan T. W. (2011). Characterization of the Asia Oceania human proteome organisation membrane proteomics initiative standard using SDS-PAGE shotgun proteomics. Proteomics 11, 4376–4384. doi: 10.1002/pmic.201100169 21887821

[B50] Perkins D. N. Pappin D. J. C. Creasy D. M. Cottrell J. S. (1999). Probability-based protein identification by searching sequence databases using mass spectrometry data. Electrophoresis 20, 3551–3567. doi: 10.1002/(sici)1522-2683(19991201)20:18<3551::Aid-elps3551>3.0.Co;2-2 10612281

[B51] Piatkov K. I. Oh J. H. Liu Y. Varshavsky A. (2014). Calpain-generated natural protein fragments as short-lived substrates of the N-end rule pathway. Proc. Natl. Acad. Sci. U. S. A. 111, E817–E826. doi: 10.1073/pnas.1401639111 24550490 PMC3948289

[B52] Picardeau M. (2017). Virulence of the zoonotic agent of leptospirosis: still terra incognita? Nat. Rev. Microbiol. 15, 297–307. doi: 10.1038/nrmicro.2017.5 28260786

[B53] Poreba M. Strozyk A. Salvesen G. S. Drag M. (2013). Caspase substrates and inhibitors. Cold Spring Harb. Perspect. Biol. 5, a008680. doi: 10.1101/cshperspect.a008680 23788633 PMC3721276

[B54] Rusu A. D. Georgiou M. (2020). The multifarious regulation of the apical junctional complex. Open Biol. 10, 190278. doi: 10.1098/rsob.190278 32070233 PMC7058937

[B55] Sato H. Coburn J. (2017). *Leptospira interrogans* causes quantitative and morphological disturbances in adherens junctions and other biological groups of proteins in human endothelial cells. PloS Negl. Trop. Dis. 11, e0005830. doi: 10.1371/journal.pntd.0005830 28750011 PMC5549773

[B56] Sato Y. Hermawan I. Kakita T. Okano S. Imai H. Nagai H.. (2022). Analysis of human clinical and environmental *Leptospira* to elucidate the eco-epidemiology of leptospirosis in Yaeyama, subtropical Japan. PloS Negl. Trop. Dis. 16, e0010234. doi: 10.1371/journal.pntd.0010234 35358181 PMC8970387

[B57] Sebastián I. Okura N. Humbel B. M. Xu J. Hermawan I. Matsuura C.. (2021). Disassembly of the apical junctional complex during the transmigration of *Leptospira interrogans* across polarized renal proximal tubule epithelial cells. Cell. Microbiol. 23, e13343. doi: 10.1111/cmi.13343 33864347 PMC8459228

[B58] Shalit T. Elinger D. Savidor A. Gabashvili A. Levin Y. (2015). MS1-based label-free proteomics using a quadrupole Orbitrap mass spectrometer. J. Proteome Res. 14, 1979–1986. doi: 10.1021/pr501045t 25780947

[B59] Sharafutdinov I. Esmaeili D. S. Harrer A. Tegtmeyer N. Sticht H. Backert S. (2020). *Campylobacter jejuni* serine protease HtrA cleaves the tight junction component claudin-8. Front. Cell. Infect. Microbiol. 10. doi: 10.3389/fcimb.2020.590186 PMC7752809 33364202

[B60] Spinelli K. J. Klimek J. E. Wilmarth P. A. Shin J. B. Choi D. David L. L.. (2012). Distinct energy metabolism of auditory and vestibular sensory epithelia revealed by quantitative mass spectrometry using MS2 intensity. Proc. Natl. Acad. Sci. U. S. A. 109, E268–E277. doi: 10.1073/pnas.1115866109 22307652 PMC3277109

[B61] Sriram S. M. Kim B. Y. Kwon Y. T. (2011). The N-end rule pathway: Emerging functions and molecular principles of substrate recognition. Nat. Rev. Mol. Cell Biol. 12, 735–747. doi: 10.1038/nrm3217 22016057

[B62] Takeichi M. (2018). Historical review of the discovery of cadherin, in memory of Tokindo Okada. Dev. Growth Differ. 60, 3–13. doi: 10.1111/dgd.12416 29278270

[B63] Toma C. Murray G. L. Nohara T. Mizuyama M. Koizumi N. Adler B.. (2014). Leptospiral outer membrane protein LMB216 is involved in enhancement of phagocytic uptake by macrophages. Cell. Microbiol. 16, 1366–1377. doi: 10.1111/cmi.12296 24655538

[B64] Toma C. Suzuki T. (2020). Evaluation of intracellular trafficking in macrophages. Methods Mol. Biol. 2134, 199–206. doi: 10.1007/978-1-0716-0459-5_18 32632871

[B65] Ueda M. Kobayashi H. Seike S. Takahashi E. Okamoto K. Yamanaka H. (2022). *Aeromonas sobria* serine protease degrades several protein components of tight junctions and assists bacterial translocation across the T84 monolayer. Front. Cell. Infect. Microbiol. 12. doi: 10.3389/fcimb.2022.824547 PMC8902146 35273923

[B66] Utech M. Mennigen R. Bruewer M. (2010). Endocytosis and recycling of tight junction proteins in inflammation. J. Biomed. Biotechnol. 2010, 484987. doi: 10.1155/2010/484987 20011071 PMC2789582

[B67] Villanueva S. Y. A. M. Saito M. Tsutsumi Y. Segawa T. Baterna R. A. Chakraborty A.. (2014). High virulence in hamsters of four dominant *Leptospira* serovars isolated from rats in the Philippines. Microbiology 160, 418–428. doi: 10.1099/mic.0.072439-0 24257815

[B68] Vu V. Light T. Sullivan B. Greiner D. Hristova K. Leckband D. (2021). P120 catenin potentiates constitutive E-cadherin dimerization at the plasma membrane and regulates trans binding. Curr. Biol. 31, 3017–3027.e7. doi: 10.1016/j.cub.2021.04.061 34019823 PMC9308376

[B69] Wieser M. Stadler G. Jennings P. Streubel B. Pfaller W. Ambros P.. (2008). hTERT alone immortalizes epithelial cells of renal proximal tubules without changing their functional characteristics. Am. J. Physiol. Renal Physiol. 295, F1365–F1375. doi: 10.1152/ajprenal.90405.2008 18715936

[B70] Yamaguchi T. Higa N. Okura N. Matsumoto A. Hermawan I. Yamashiro T.. (2018). Characterizing interactions of *Leptospira interrogans* with proximal renal tubule epithelial cells. BMC Microbiol. 18, 64. doi: 10.1186/s12866-018-1206-8 29973159 PMC6030750

[B71] Yap A. S. Gomez G. A. Parton R. G. (2015). Adherens junctions revisualized: Organizing cadherins as nanoassemblies. Dev. Cell 35, 12–20. doi: 10.1016/j.devcel.2015.09.012 26460944

[B72] Yates J. R. Eng J. K. McCormack A. L. Schieltz D. (1995). Method to correlate tandem mass spectra of modified peptides to amino acid sequences in the protein database. Anal. Chem. 67, 1426–1436. doi: 10.1021/ac00104a020 7741214

