# Supplementary figures and images for: Degradation of p0071 and p120-catenin during adherens junction disassembly by Leptospira interrogans
Source: Front Cell Infect Microbiol. 2023 Sep 15;13:1228051. doi: 10.3389/fcimb.2023.1228051 (PMC10545952; doi:10.3389/fcimb.2023.1228051)

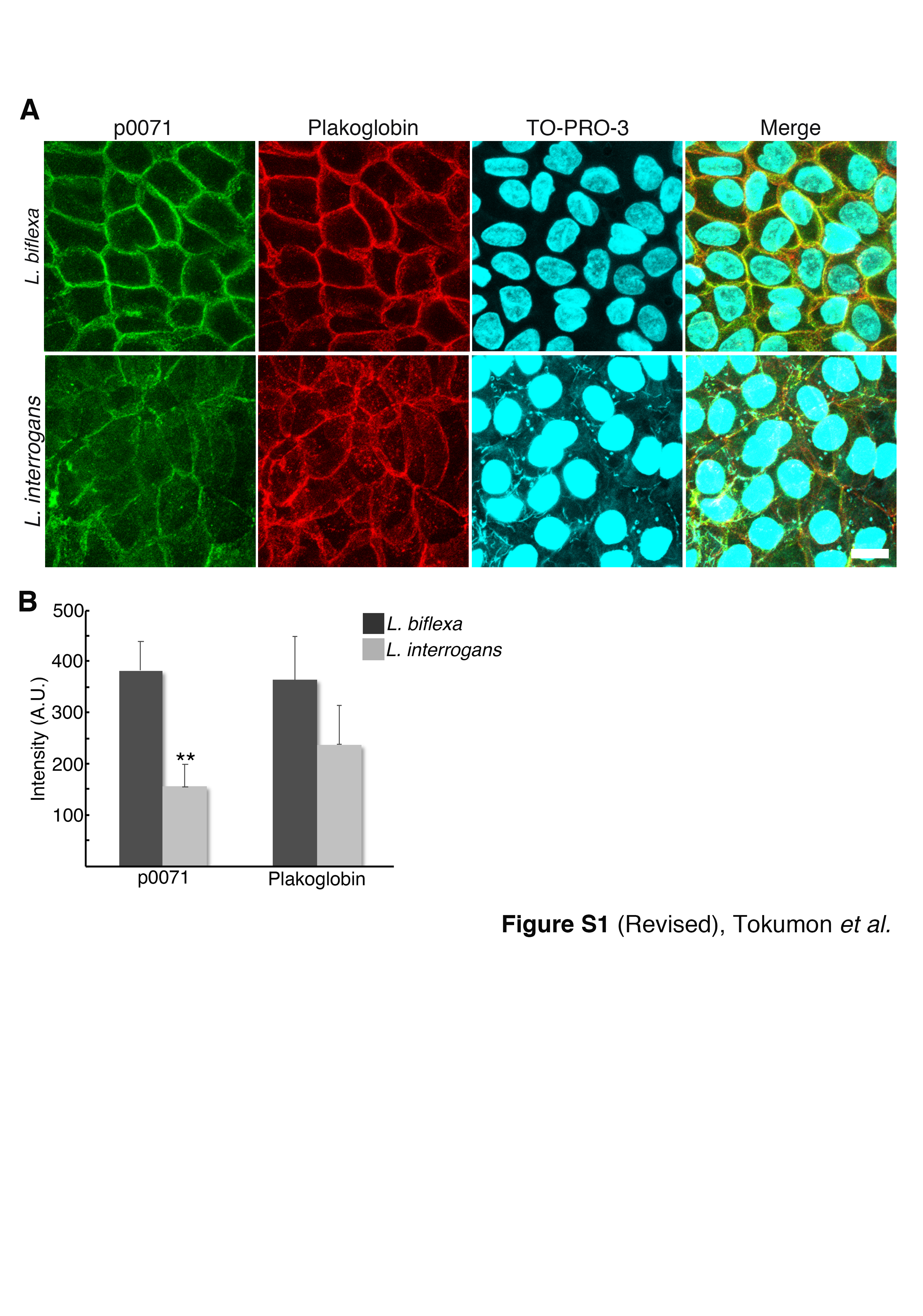

Supplement: Supplementary Figure 1 — L. interrogans induces aberrant p0071 localization and partial plakoglobin mislocalization. (A) Representative confocal images of L. biflexa or L. interrogans-infected RPTECs at 24 h p.i. p0071 was stained with an Alexa Fluor 488-labeled antibody (green), and plakoglobin was stained with a Cy3-labeled antibody (red). The cell nuclei and leptospiral DNA were counterstained with TO-PRO-3 (cyan). Scale bar: 10 μm. (B) Quantification of fluorescence intensity at cell-cell junctions. Each bar represents the mean ± standard deviation of three independent experiments. A.U.: arbitrary units. **p <0.01. [file Image_1.tif]

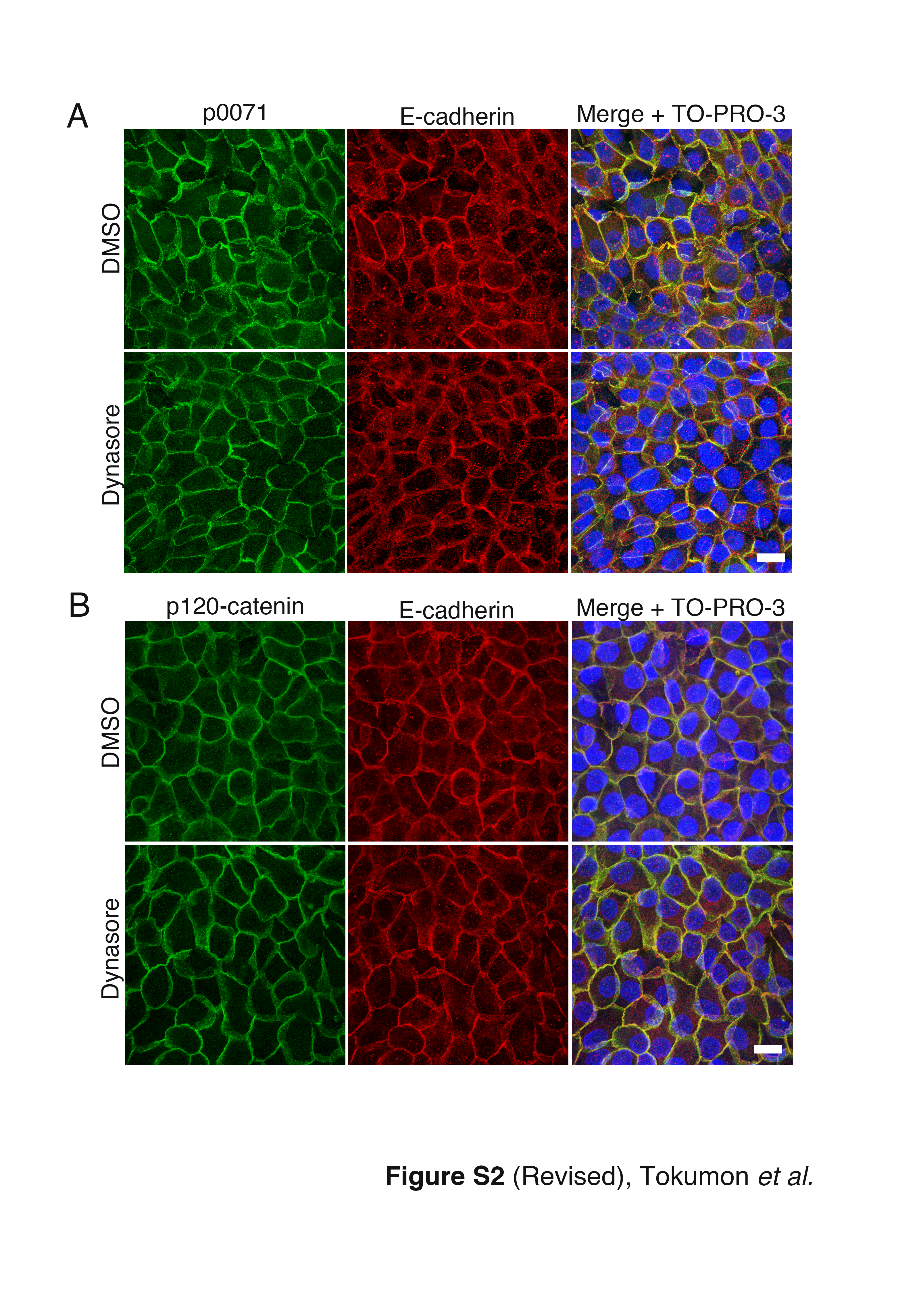

Supplement: Supplementary Figure 2 — Dynasore does not affect p0071 and p120-ctn localization in non-infected RPTECs. DMSO- or Dynasore-pre-treated RPTECs were fixed and processed for immunofluorescence analysis. RPTECs were immunostained with anti-p0071 (A) or p120-ctn (B) antibodies and then visualized with Alexa Fluor 488-conjugated secondary antibodies (green). E-cad was detected using a Cy3-labeled antibody (red) and the cell nuclei were detected with TO-PRO-3 (blue). Scale bar: 10 μm. [file Image_2.tif]

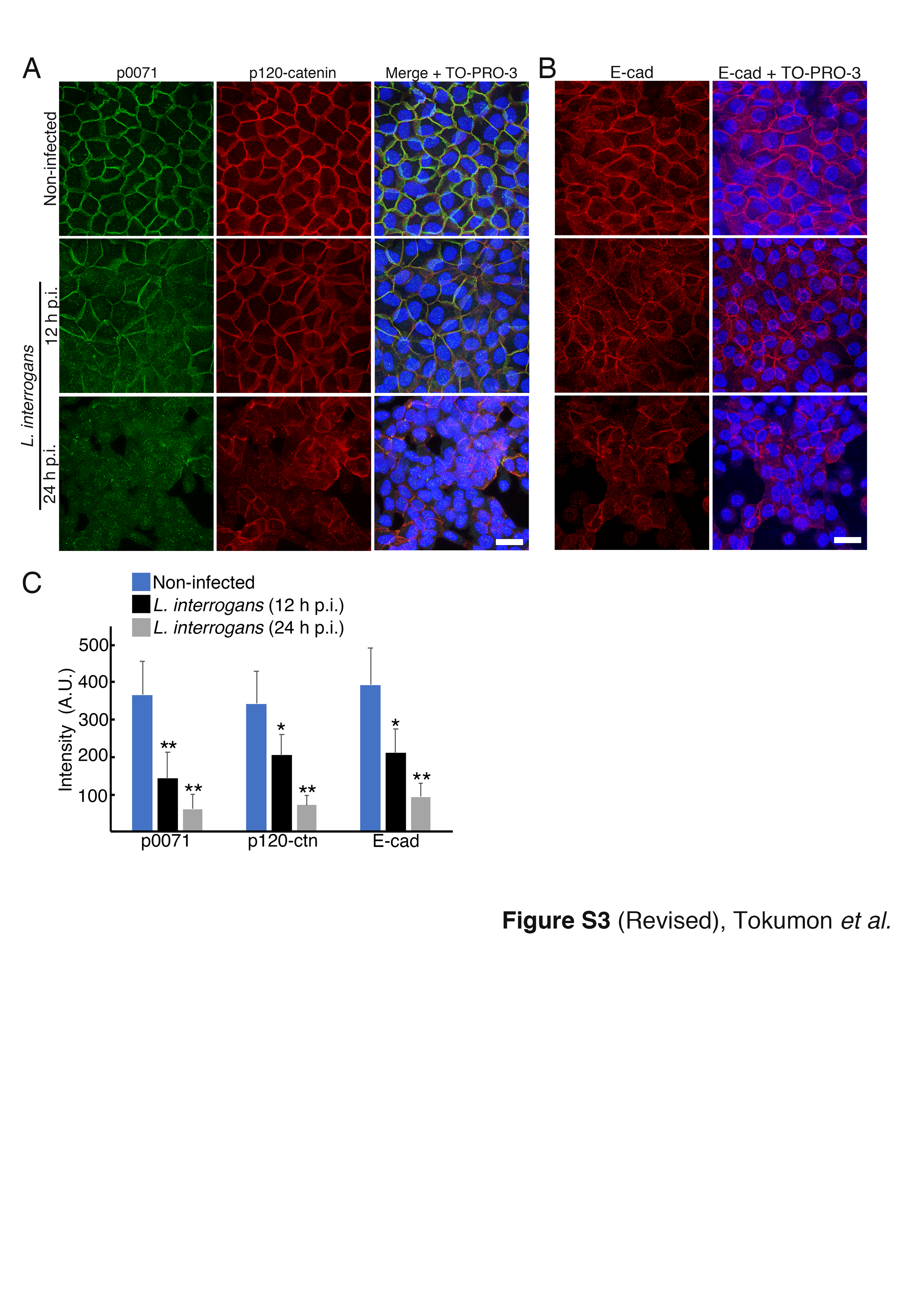

Supplement: Supplementary Figure 3 — Dynamics of E-cad, p0071, and p120-ctn localization at AJs during L. interrogans infection of RPTECs. (A, B) Representative confocal images of non-infected or L. interrogans-infected RPTECs. Cells were methanol fixed and processed for immunostaining at 12 h and 24 h p.i. (A) p0071 was stained with an Alexa Fluor 488-labeled antibody (green), and p120-ctn was stained with a Cy3-labeled antibody (red). (B) E-cad was stained with an Cy3-labeled antibody (red). (A, B) The cell nuclei were counterstained with TO-PRO-3 (blue). Scale bar: 10 μm. (C) Quantification of fluorescence intensity at cell-cell junctions. Each bar represents the mean ± standard deviation of three independent experiments. A.U.: arbitrary units. *p <0.05 and **p <0.01. [file Image_3.tif]

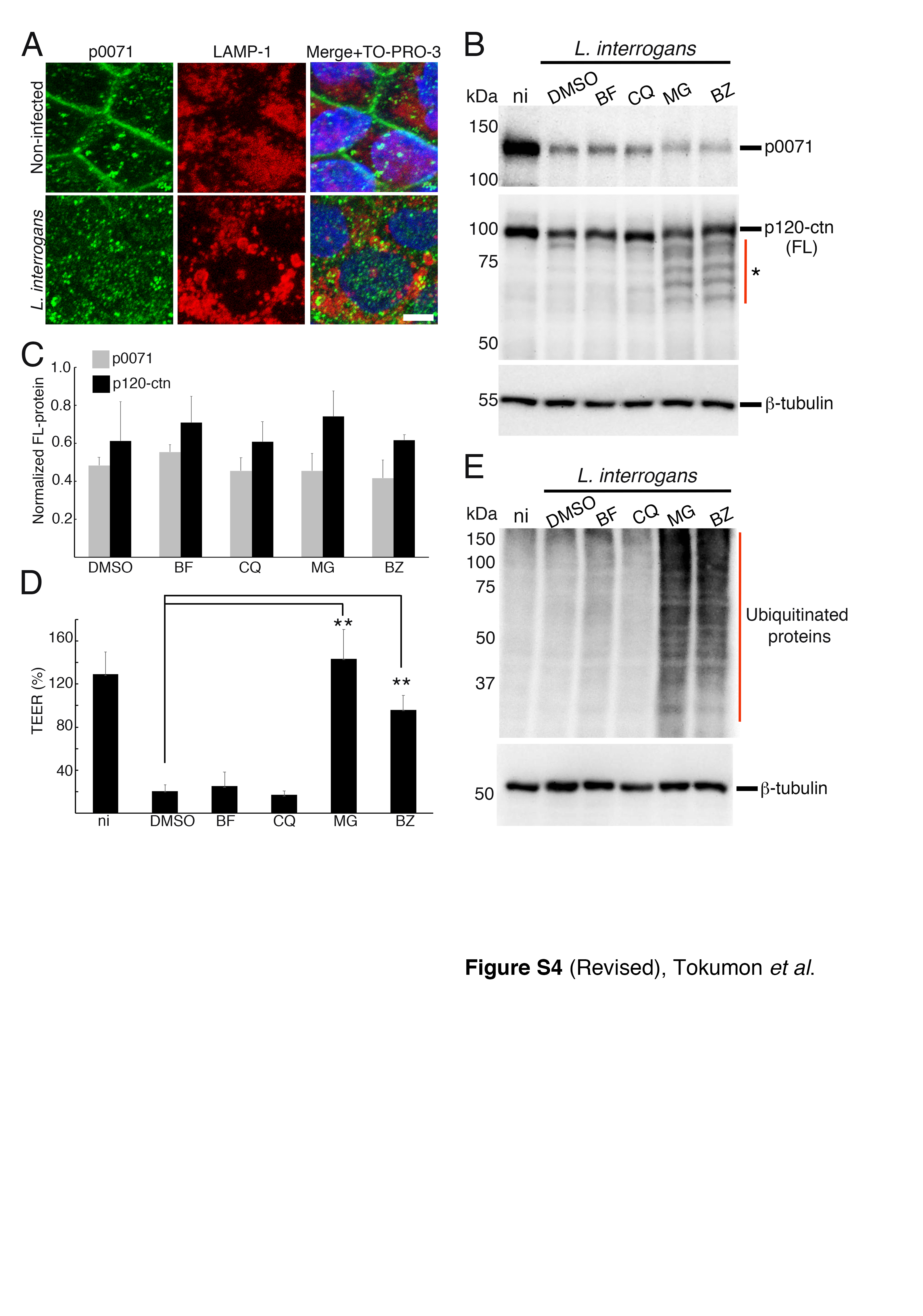

Supplement: Supplementary Figure 4 — Lysosomal and proteasomal inhibitors are unable to prevent p0071 and p120-ctn degradation. (A) Dual immunofluorescence staining to detect p0071 (green) and the lysosomal associated membrane protein (LAMP-1, red) in non-infected and L. interrogans-infected RPTECs at 24 h p.i. Scale bar: 5 μm. (B) RPTECs were pre-treated for 30 min with either the lysosomal inhibitors (bafilomycin A1 (BF) or chloroquine (CQ)) or the proteasomal inhibitors (MG-132 (MG) or bortezomib (BZ)) and infected with L. interrogans. Whole-cell lysates were subjected to western blotting at 18 h p.i. Fragments that were newly detected by adding inhibitors are denoted by an asterisk. (C) Normalized levels of full-length (FL)-proteins. Each bar represents the mean ± standard deviation of three independent experiments. (D) Transepithelial electrical resistance (TEER) measurements at 18 h p.i., **p <0.01. (E) Detection of total ubiquitinated-proteins in L. interrogans-infected RPTECs after the addition of the inhibitors as described in (B). [file Image_4.tif]

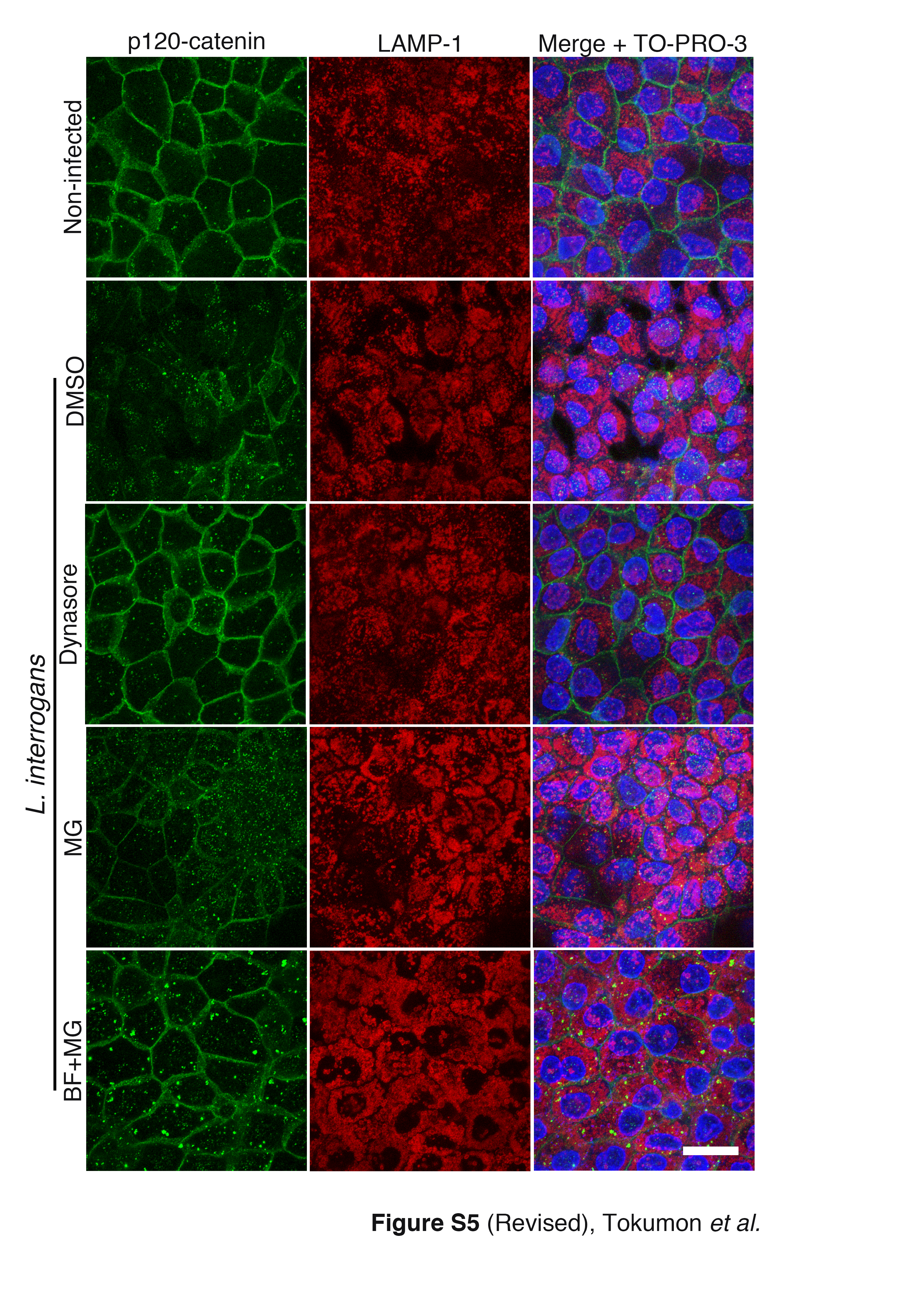

Supplement: Supplementary Figure 5 — p120-ctn localization in LAMP-1-positive vacuoles. RPTECs were pre-treated for 30 min with DMSO, dynasore, MG, or BF+MG; infected with L. interrogans for 18 h; and processed for immunofluorescence. p120-ctn was stained with an Alexa Fluor 488-labeled antibody (green), while LAMP-1 was stained with a Cy3-labeled antibody (red). The cell nuclei were stained with TO-PRO-3 (blue). Scale bar: 20 μm. [file Image_5.tif]

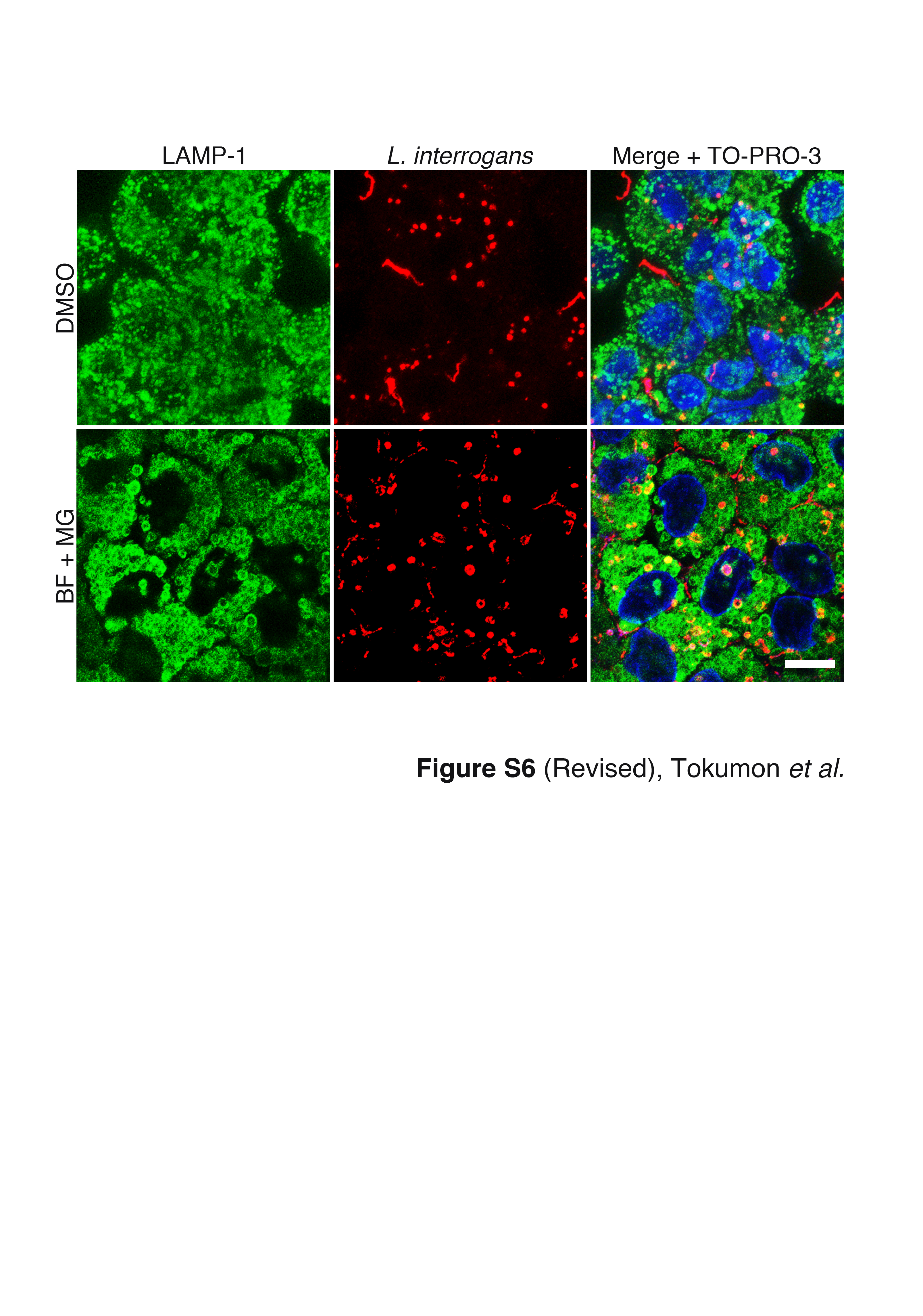

Supplement: Supplementary Figure 6 — L. interrogans localizes in LAMP-1-positive vacuoles. RPTECs were pre-treated with DMSO or BF+MG and infected with L. interrogans. Representative confocal images showing infected RPTECs (18 h p.i.). LAMP-1 was stained with an Alexa Fluor 488-labeled antibody (green), while L. interrogans was stained with a Cy3-labeled antibody (red). The cell nuclei were stained with TO-PRO-3 (blue). Scale bar: 10 μm. [file Image_6.tif]

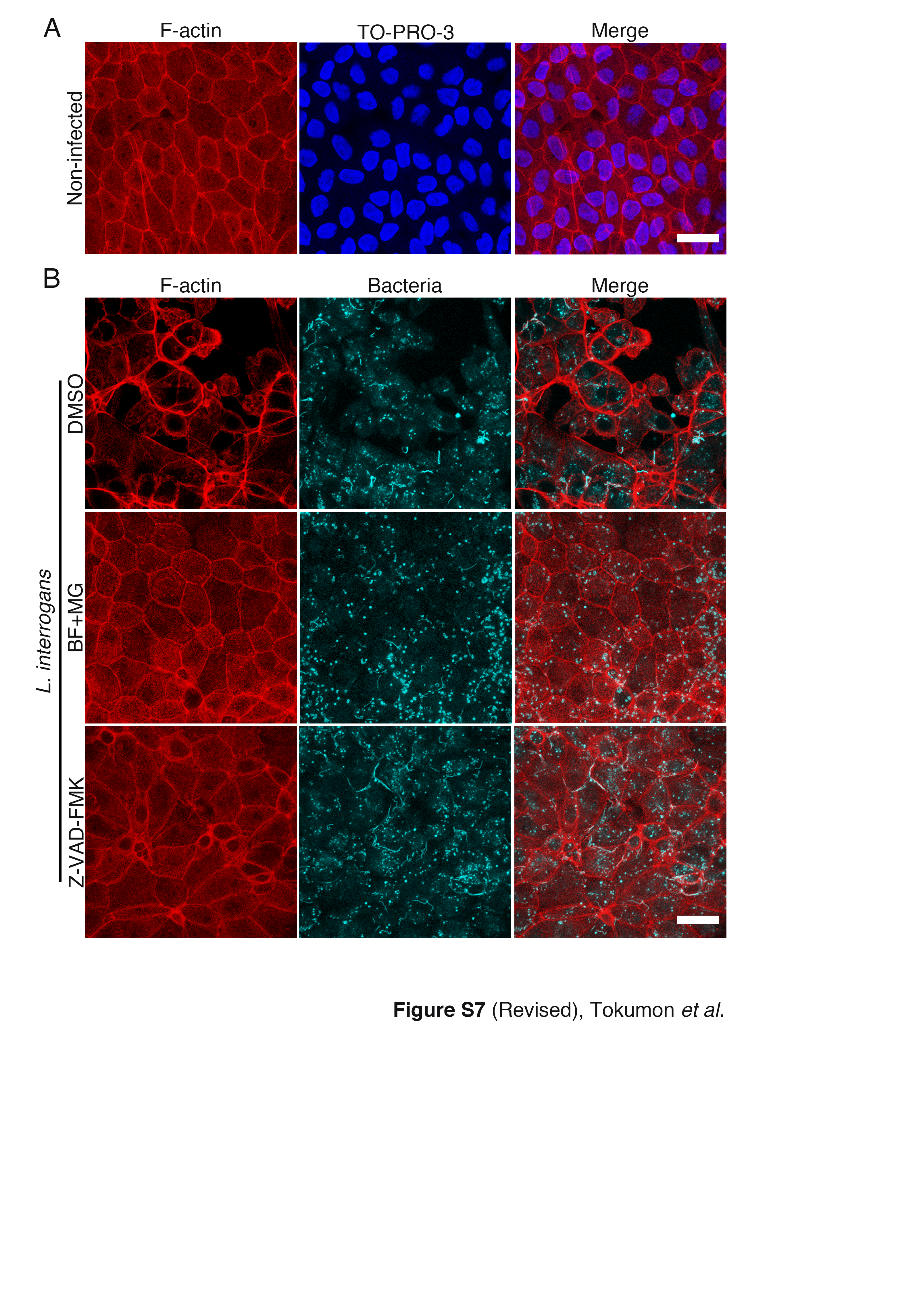

Supplement: Supplementary Figure 7 — Z-VAD-FMK partially prevents L. interrogans-induced F-actin rearrangement. Representative confocal images of (A) non-infected RPTECs or (B) L. interrogans-infected RPTECs. (B) RPTECs were pre-treated for 30 min with DMSO, BF+MG, or Z-VAD-FMK and infected with L. interrogans for 24 h. L. interrogans was stained with an Alexa 647-labeled antibody (cyan) and F-actin with rhodamine-phalloidin (red). The cell nuclei were stained with TO-PRO-3 (blue) in (A). Scale bars: 20 μm. [file Image_7.tif]
